# Meta-Review of the Quantity and Quality of Evidence for Knee Arthroplasty Devices

**DOI:** 10.1371/journal.pone.0163032

**Published:** 2016-10-03

**Authors:** Anna R. Gagliardi, Ariel Ducey, Pascale Lehoux, Sue Ross, Patricia Trbovich, Anthony Easty, Chaim Bell, Julie Takata, Christof Pabinger, David R. Urbach

**Affiliations:** 1 University Health Network, Toronto, Canada; 2 University of Calgary, Calgary, Canada; 3 University of Montreal, Montreal, Canada; 4 University of Alberta, Edmonton, Canada; 5 University of Toronto, Toronto, Canada; 6 Mount Sinai Hospital, Toronto, Canada; 7 Medical University of Innsbruck, Innsbruck, Austria; Cardiff University, UNITED KINGDOM

## Abstract

**Introduction:**

Some cardiovascular devices are licensed based on limited evidence, potentially exposing patients to devices that are not safe or effective. Research is needed to ascertain if the same is true of other types of medical devices. Knee arthroplasty is a widely-used surgical procedure yet implant failures are not uncommon. The purpose of this study was to characterize available evidence on the safety and effectiveness of knee implants.

**Methods:**

A review of primary studies included in health technology assessments (HTA) on total (TKA) and unicompartmental knee arthroplasty (UKA) was conducted. MEDLINE, EMBASE, CINAHL, Cochrane Library and Biotechnology & BioEngineering Abstracts were searched from 2005 to 2014, plus journal tables of contents and 32 HTA web sites. Patients were aged 18 and older who underwent primary TKA or UKA assessed in cohort or randomized controlled studies. Summary statistics were used to report study characteristics.

**Results:**

A total of 265 eligible primary studies published between 1986 and 2014 involving 59,217 patients were identified in 10 HTAs (2 low, 7 moderate, 1 high risk of bias). Most evaluated TKA (198, 74.5%). The quality of evidence in primary studies was limited. Most studies were industry-funded (23.8%) or offered no declaration of funding or conflict of interest (44.9%); based on uncontrolled single cohorts (58.5%), enrolled fewer than 100 patients (66.4%), and followed patients for 2 years or less (UKA: single cohort 29.8%, comparative cohort 16.7%, randomized trial 25.0%; TKA: single cohort 25.0%, comparative cohort 31.4%, randomized trial 48.6%). Furthermore, most devices were evaluated in only one study (55.3% TKA implants, 61.1% UKA implants).

**Conclusions:**

Patients, physicians, hospitals and payers rely on poor-quality evidence to support decisions about knee implants. Further research is needed to explore how decisions about the use of devices are currently made, and how the evidence base for device safety and effectiveness can be strengthened.

## Introduction

Medical decision-making is meant to be informed by the best available evidence, clinical judgment, and patient values and preferences. However it appears that what constitutes evidence may differ between drug and non-drug technologies. While pharmaceutical products must undergo years of rigorous testing, analysis of evidence for high-risk cardiovascular devices approved by the United States Food and Drug Administration found that the quantity and quality of pre- and post-market studies was lacking, potentially exposing patients to devices that were not safe and effective [1,2]. The same was true of metal-on-metal total hip replacement, which was associated with high revision rates, and subsequent analysis of explanted components found that they had been modified from the manufacturer’s specifications [3].

Before advocating for broad changes in the policies and processes of pre- and/or post-market surveillance, further studies are needed to ascertain if the same is true of other types of medical devices. Knee arthroplasty is among the most common and effective procedure currently performed [4], and is expected to increase in frequency [5]. However, surgical complications and implant failures are not uncommon, with ten-year revision rates of 6.2% for total (TKA), and 16.5% for unicompartmental knee arthroplasty (UKA) [6,7].

No research has fully described the evidence that payers, hospitals, physicians and patients must rely on when making decisions about knee arthroplasty. It was hypothesized that, similar to studies of cardiovascular devices [1–3], evidence on the safety and effectiveness of knee implants may be limited due to issues of randomization, blinding, and the expense of measuring long-term outcomes [5]. The purpose of this study was to characterize the nature of the available evidence regarding the safety and effectiveness of knee arthroplasty devices. Specifically, this study sought to describe limitations of studies that evaluated knee implants.

## Methods

### Approach

This study described the limitations of studies that evaluated knee arthroplasty devices; it did not seek to assess if knee implants are clinically effective as that research has been done [4]. A meta-review was conducted of primary studies included in health technology assessments (HTAs) of knee arthroplasty devices. HTA is defined as the systematic evaluation of properties, effects and/or impacts of health technologies and interventions including intended and unintended consequences [6]. We used the Preferred Reporting Items for Systematic Reviews and Meta-Analyses (PRISMA) criteria (S1 Checklist) [7]. A protocol for this review was not registered. Institutional review board approval was not necessary.

### Eligibility

Inclusion and exclusion criteria were initially generated based on the Patients, Intervention, Comparison and Outcomes (PICO) framework, and used to search and screen for HTAs, and then to screen primary studies included in those reviews. Patients included adults aged 18 and older from any country who underwent knee arthroplasty for any indication. The intervention of interest was primary total (TKA) or unicompartmental knee arthroplasty (UKA). Comparisons included single cohort studies evaluating a device, either before and after, or only after surgery; or comparative cohort studies or randomized trials comparing patients before and after, or only after receiving different types of devices. Studies varied in the outcomes they reported. To include studies that evaluated the safety and effectiveness of devices while favouring inclusion, eligible studies reported at least two of the following most frequently reported outcomes: complications (surgical or device-specific), revision rate (absolute number or device survival), or functional outcomes (e.g. pain, health status, quality of life, ability to complete physical tasks, satisfaction) assessed either by clinicians or patients using standardized instruments. Searches were limited to English language. Publications in the form of editorials, protocols, abstracts, or proceedings were not eligible. Studies were not eligible if they focused on evaluating the effectiveness of a surgical approach (i.e. minimally invasive, computer-aided) or technique (e.g. posterior, lateral or anterior approach; type of incision, sutures, instrumentation, bone cement), or on rehabilitation interventions or quality of life following surgery. This review focused on HTAs because HTAs are a form of evidence that is readily available to the majority of health care professionals to inform real-time decisions about which devices to purchase and use; and include primary studies of pre- and post-market evaluation upon which regulatory licensing decisions are made. Studies based on registry data were excluded. Although such studies provide useful data due to the large number of included patients, they do so only after a considerable period of time during which devices were licensed and used in many patients.

### Searching and screening

Knee arthroplasty HTAs were identified in MEDLINE, EMBASE, CINAHL, Health Technology Assessment database in the Cochrane Library, and Biotechnology & BioEngineering Abstracts. These were searched on January 14, 2015 from 2005 to 2014 inclusive. The search strategy (S1 Table) was purposefully broad to be as inclusive as possible. We also searched the tables of contents of Health Technology Assessment and the International Journal of Technology Assessment in Health Care, and 35 web sites of HTA agencies (S2 Table). Titles and abstracts were independently screened by three reviewers. All items selected by at least one reviewer were retrieved. Then the primary studies included in HTAs were screened. If two or more primary studies were based on the same cohort of patients, the single most recent or complete study was eligible and the outcomes it reported were included.

### Data extraction

Data were extracted from primary studies on author, country, year published, HTA source, arthroplasty type (UKA, TKA) and device (model, company). To identify limitations of the studies that evaluated knee implants, data were extracted on study design, number of patients included in final analyses, years of follow-up and conflict of interest (independent, industry funded, undetermined). We did not extract outcome data; the effectiveness of knee arthroplasty has already been established (4). ARG and two trained research assistants independently pilot-tested data extraction on the same three articles and compared findings through two iterations at which time data extraction was congruent. Two research assistants extracted data from remaining studies. ARG independently checked data to resolve discrepancies or other issues.

### Data analysis

The methodological quality of HTAs was assessed using the Assessing the Methodological Quality of Systematic Reviews (AMSTAR) instrument [8]. Each study was scored for the presence of 11 elements, and the total score was categorized as high (0 to 4), moderate (5 to 8) and low (9 to 11) risk of bias. Summary statistics were used to describe the number of studies by country, year of publication, and type of implant. The methodological quality of primary studies was described with summary statistics for study design, number of participants, length of follow-up and potential conflicts of interest.

## Results

### Search results

**Fig 1:**
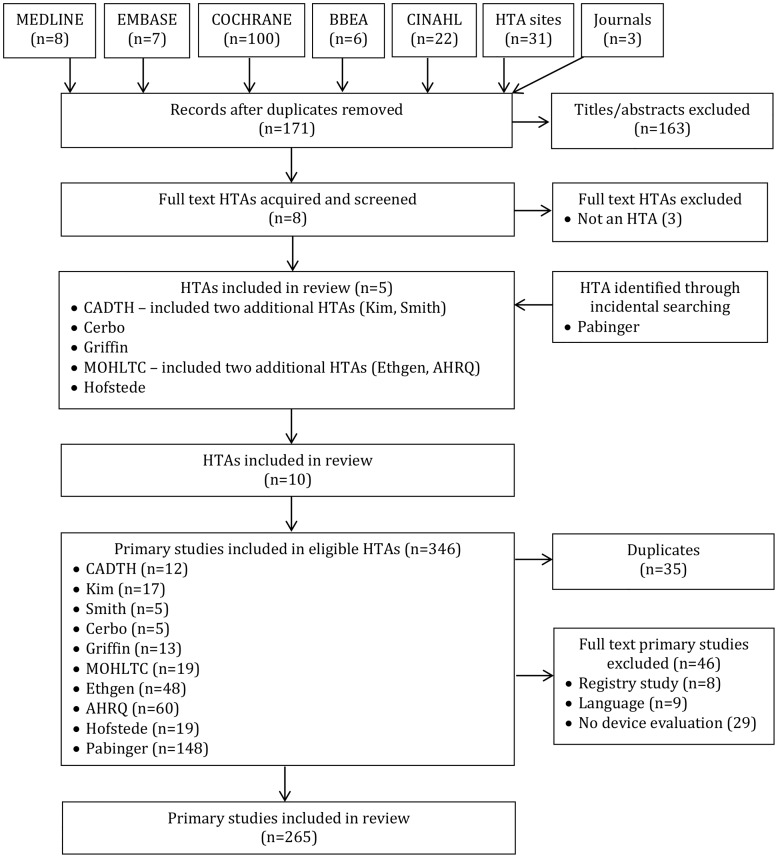
PRISMA diagram.

The PRISMA diagram appears in Fig 1. Ten HTAs were eligible [9–19]. Those HTAs included 346 primary studies, of which 35 were duplicates, 46 were excluded (29 no device evaluation, 9 non-English language, 8 registry studies), leaving 265 primary studies that were included in the review.

### HTA characteristics

Of the 10 HTAs, 2 had a low risk of bias, 7 had a moderate risk of bias and 1 had a high risk of bias (Table 1, based on S3 Table). Two HTAs were issued in Canada and the United States, and one each in Austria, Australia, Belgium, Italy, Netherlands, and the United Kingdom. HTAs included a median of 15.5 studies (range 3 to 115).

**Table 1:** Design of studies included in eligible health technology assessments.

Author, [ref], Year, Country	AMSTAR Risk of bias	Knee system	Time span	Design of eligible studies (n, % of eligible studies by row)			Eligible studies
				RCT	CC	SC	
Pabinger [9,10], 2015, Austria	10 (Low)	TKA, UKA	1986 to 2009	11 (9.6)	28 (24.3)	76 (66.1)	115
Hofstede [11], 2015, Netherlands	11 (Low)	TKA	2001 to 2014	15 (100.0)	0 (0.0)	0 (0.0)	15
Kim [12], 2014, USA	5 (Moderate)	TKA, UKA	2004 to 2012	1 (6.3)	1 (6.3)	14 (87.5)	16
CADTH [13], 2013, Canada	5 (Moderate)	TKA, UKA	2009 to 2013	2 (16.7)	0 (0.0)	10 (83.3)	12
Smith [14], 2009, UK	7 (Moderate)	UKA	2002 to 2007	3 (60.0)	2 (40.0)	0 (0.0)	5
Cerbo [15], 2009, Italy	7 (Moderate)	TKA	1998 to 2007	2 (66.7)	0 (0.0)	1 (33.3)	3
Griffin [16], 2005, Australia	7 (Moderate)	TKA, UKA	1988 to 2004	0 (0.0)	3 (37.5)	5 (62.5)	8
Medical Advisory Secretariat [17], 2005, Canada	6 (Moderate)	TKA	2003 to 2005	2 (10.5)	4 (21.1)	13 (68.4)	19
Ethgen [18], 2004, Belgium	4 (High)	TKA, UKA	1994 to 2001	0 (0.0)	3 (18.8)	13 (81.3)	16
Kane [19], 2003, USA	7 (Moderate)	TKA	1995 to 2003	5 (8.9)	21 (37.5)	30 (53.6)	56
Total				41 (15.5)	62 (23.4)	162 (61.1)	265

AMSTAR A Measurement Tool to Assess Systematic Reviews (high 0–4, moderate 5–8, low risk of bias 9–11).

UKA unicompartmental knee arthroplasty; TKA total knee arthroplasty.

SR systematic review, meta-analysis or health technology assessment; RCT randomized controlled trial; CC comparative cohort study; SC single cohort study, either retrospective, prospective or before/after.

### Primary study characteristics

The 265 primary studies were published between 1986 and 2014 by authors from 28 countries.

The majority of studies were published by authors in the United States (101/265, 38.1%). Among the 265 studies, 63 (23.8%) declared industry funding, 83 (31.3%) declared independent funding, and 119 (44.9%) offered no explicit conflict of interest statement or acknowledgement of funding.

### Devices evaluated

The majority of devices were evaluated in very few studies (Table 2). For example, 26 of 47 (55.3%) TKA implants and 11 of 18 (61.1%) UKA implants were each evaluated in a single study.

**Table 2:** Implants evaluated in eligible studies.

Type and brand of device	Studies reporting number of patients (n)							Studies NR patients (n)
	Studies	Study Design			Patients*			
		SC	CC	RCT	Explicit	Unclear (≤)	Min/Max (estimate)	
TKA								
Press Fit Condylar (DePuy/Johnson&Johnson)	47	25	11	11	6,409	601	7,010	1
Anatomic Graduated Component (Biomet)	32	16	11	5	20,942	2,087	23,029	3
Low Contact Stress (DePuy/Johnson&Johnson)	30	19	5	6	3,333	462	3,795	2
Kinemax (Howmedica)	15	9	6	0	3,006	162	3,168	1
Insall-Burstein (Zimmer)	9	4	4	1	229	1,963	2,192	3
Kinematic (Howmedica)	8	5	2	1	230	1,259	1,489	0
Total Condylar (Howmedica)	7	6	1	0	409	405	814	0
NexGen (Zimmer)	7	2	2	3	586	212	798	0
Genesis (Smith & Nephew)	6	0	4	2	776	65	841	0
Anatomic Modular Knee (DePuy/Johnson&Johnson)	6	0	2	4	294	335	629	0
Porous Coated Anatomic (Howmedica)	4	2	2	0	575	192	767	0
Miller-Galante (Zimmer)	4	2	2	0	212	92	304	0
e.motion (B.Braun Aesculap)	2	0	1	1	0	124	124	0
Oxford (Biomet)	2	1	0	1	96	55	151	0
Total Meniscal Knee (Biomet)	2	1	0	1	33	171	204	0
Nuffield Knee (Corin Medical)	2	0	1	1	0	51	51	1
Rotaglide (Corin Medical)	2	0	0	2	0	72	72	0
Synatomic (DePuy)	2	1	1	0	75	70	145	0
Duracon Total Knee (Howmedica)	2	2	0	0	201	0	201	0
Natural Knee (Intermedics Orthopedics)	2	1	1	0	469	0	469	0
St. Georg Sled (Waldemar Link)	2	2	0	0	35	414	449	0
Columbus CR (B.Braun Aesculap)	1	0	0	1	0	99	99	0
Columbus RP (B.Braun Aesculap)	1	0	0	1	0	99	99	0
Search (B.Braun Aesculap)	1	0	1	0	125	0	125	0
Maxim Complete (Biomet)	1	0	1	0	240	0	240	0
Vanguard (Biomet)	1	0	1	0	0	205	205	0
Anatomic Medullary Knee (DePuy)	1	0	0	1	100	0	100	0
Whiteside Ortholoc (Dow Corning)	1	0	1	0	0	87	87	0
Foundation Knee (Encore)	1	0	0	1	0	79	79	0
Duopatellar (Johnson & Johnson)	1	1	0	0	0	47	47	0
Total Condylar III (Johnson & Johnson)	1	1	0	0	0	45	45	0
Multigen Plus (Lima)	1	0	0	1	0	118	118	0
BalanSys (Mathys Medical)	1	0	0	1	92	0	92	0
Minns Meniscal Knee (NR)	1	1	0	0	26	0	26	0
St. Leger (NR)	1	0	1	0	0	33	33	0
Osteonics (Omnifit)	1	0	1	0	0	87	87	0
Freeman-Samuelson (Protek)	1	1	0	0	120	0	120	0
Imperial College London Hospital (Protek)	1	0	1	0	0	19	19	0
Tricon M (Richards Manufacturing)	1	0	1	0	0	19	19	0
Trekking (Samo)	1	0	0	1	0	118	118	0
Scorpio (Stryker)	1	0	0	1	81	0	81	0
Total Articulating Cementless Knee (WaldemarLink)	1	1	0	0	102	0	102	0
Medial Pivot (Wright Medical)	1	0	0	1	0	91	91	0
UKA								
Oxford (Biomet)	42	33	5	4	5,410	544	5,954	2
St. Georg Sled (Waldemar Link)	9	5	2	2	577	1,500	2,077	0
Low Contact Stress (DePuy)	3	3	0	0	100	96	196	1
Miller-Galante (Zimmer)	3	1	1	1	75	146	221	0
Preservation (DePuy)	3	2	1	0	132	0	132	0
Press Fit Condylar (DePuy)	2	1	1	0	15	99	114	0
Robert Brigham (DePuy/Johnson&Johnson)	3	2	1	0	18	134	152	0
AMC Unicondylar (Alphanorm)	1	0	0	1	0	39	39	0
Avon (Stryker)	1	1	0	0	29	0	29	0
Fixed Allegretto (Centerpulse)	1	0	0	1	0	39	39	0
HLS Prosthesis (Tornier)	1	0	1	0	221	0	221	0
Imperial College London Hospital (Protek)	1	0	1	0	0	19	19	0
Journey (Smith & Nephew)	1	1	0	0	22	0	22	0
Porous Coated Anatomic (Howmedica)	1	1	0	0	0	9	9	0
Replicci Implant (Biomet)	1	0	1	0	N/A	N/A	N/A	1
Tricon M (Richards Manufacturing)	1	0	1	0	0	19	19	0
Tricon P (Richards Manufacturing)	1	0	1	0	0	19	19	0
Unicondylar (B.Braun Aesculap)	1	1	0	0	28	0	28	0

N/A not applicable; SC single cohort; CC comparative cohort; RCT randomized controlled trial; NR not reporting.

* Notes regarding number of reported patients in eligible studies; categories are mutually exclusive:

Explicit—the number of participating patients was clearly reports

Unclear—studies used two or more devices but did not report the division of patients/knees between these thus the number reported here represents the total number of patients (i.e. 50 knees were implanted with either Device X or Device Y)

Min/Max—studies reported either a minimum or maximum number of participants; the number reported here reflects the number of participants reported by studies that stated either a minimum or maximum

### Study design

Most studies evaluated TKA (198, 74.5%) (Table 3). Overall, the majority of studies were based on single cohorts (SC, 162, 61.1%), followed by comparative cohorts (CC, 62, 23.4%) and randomized controlled trials (41, 15.5%).

**Table 3:** Number of patients evaluated in eligible studies.

Study design (n, % of total)	Knee system	Studies (n)		Sample size by percentile (n)					Sample size by study design and knee system (n)	Total number of patients by study design (n, % of total)
		Reported	NR or unclear	0 (min)	25	50 (median)	75	100 (max)		
SC (162, 61.1)	TKA	108	4	14.0	62.0	105.5	208.3	4,393.0	30,102	36,488 (61.6)
	UKA	44	3	10.0	33.8	73.0	126.0	881.0	5,826	
	Both	3	0	48.0	72.5	97.0	256.0	415.0	560	
CC (62, 23.4)	TKA	47	4	12.0	94.5	125.0	252.5	3,998.0	16,623	18,775 (31.7)
	UKA	5	1	28.0	53.0	88.0	221.0	447.0	837	
	Both	5	0	20.0	100.0	206.0	239.0	750.0	1,315	
RCT (41, 15.5)	TKA	35	0	20.0	43.0	92.0	114.5	390.0	3,634	3,954 (6.7)
	UKA	4	0	40.0	46.0	55.0	69.5	92.0	242	
	Both	2	0	22.0	30.5	39.0	47.5	56.0	78	
Total N = 265	All	253	12	10.0	56.0	100.0	194.0	4,393.0	59,217	59,217

NR not reported; SC single cohort; CC comparative cohort; RCT randomized controlled trial.

### Participants

Across 265 studies, 59,217 patients were evaluated (Table 3). Notably, several studies failed to report the number of patients who received knee implants. Overall, most studies had 100 or fewer participants. By study design, 61.6% of patients were assessed in SC with a median (range) of 105.5 (14.0 to 4,393.0) patients in all TKA studies; 73.0 (10.0 to 881.0) patients in all UKA studies; and 97.0 (48.0 to 415.0) patients across all studies that evaluated both types of implants. This was followed by 31.7% of patients assessed in CC (TKA median 125.0, range 12.0 to 3,998.0; UKA median 88.0, range 28.0 to 447.0; both median 206.0, range 20.0 to 750.0); and 6.7% of patients assessed in RCTs (TKA median 92.0, range 20.0 to 390.0; UKA median 55.0, range 40.0 to 92.0; both median 39.0, range 22.0 to 56.0).

### Follow-up period

Patients were followed for 2 years or less in a high proportion of studies for both UKA (SC 29.8%, CC 16.7%, RCT 25.0%) and TKA (25.0%, 31.4%, 48.6%) (Table 4)

**Table 4:** Patient follow-up period in eligible studies by study design and knee system.

Study design (n, % of total)	Knee system (n, % by study design)	Follow-up period (n, % by type of implant)		
		≤ 2 years	> 2 years	Not reported or unclear
SC (162, 61.1)	TKA (112, 69.1)	28 (25.0)	79 (70.5)	5 (4.5)
	UKA (47, 29.0)	14 (29.8)	32 (68.1)	1 (2.1)
	Both (3, 1.9)	0 (0.0)	2 (66.7)	1 (33.3)
CC (62, 23.4)	TKA (51, 82.3)	16 (31.4)	34 (66.7)	1 (2.0)
	UKA (6, 9.7)	1 (16.7)	5 (83.3)	0 (0.0)
	Both (5, 8.1)	4 (80.0)	1 (20.0)	0 (0.0)
RCT (41, 15.5)	TKA (35, 85.4)	17 (48.6)	18 (51.4)	0 (0.0)
	UKA (4, 9.8)	1 (25.0)	3 (75.0)	0 (0.0)
	Both (2, 4.9)	0 (0.0)	2 (100.0)	0 (0.0)
Total (265)	(265)	81 (30.6)	176 (66.4)	8 (3.0)

SC single cohort; CC comparative cohort; RCT randomized controlled trial.

## Discussion

While hundreds of studies on knee arthroplasty devices were identified, there is little reliable data on the effectiveness and safety of most types of knee implants. Of 265 eligible primary studies, the findings of 70% of the primary studies that were industry- or undeclared sponsorship should be interpreted with some caution. The quality of evidence in primary studies was limited. Most studies were based on uncontrolled single cohorts, enrolled fewer than 100 patients, and followed patients for 2 years or less. Furthermore, most devices were evaluated in only one study. If safety or effectiveness of devices is a key concern, decisions regarding the choice of medical devices appear to be largely unsupported by reliable evidence.

Similar findings were identified in other assessments of syntheses and of primary studies. Sharma et al. [20] assessed the methodological quality of 77 meta-analyses in joint arthroplasty. Among these5 (6%) had extensive flaws, 34 (44%) had major flaws, 30 (39%) had minor flaws, and 8 (10%) had minimal flaws; the quality of 14 meta-analyses based on TKA was not reported. Nieuwenhuijse et al. [21] conducted a systematic review to appraise the evidence base for orthopedic devices including high flexion TKA. Among 56 studies describing 52 cohorts, study quality was judged to be low or moderate in over 60% of 56 studies describing 52 cohorts. However, our review was a more comprehensive assessment of the quality of primary studies on knee implants than either of these studies. Our meta-review included fewer syntheses of knee arthroplasty than the Sharma et al. study [20], but it included more detail about the quality of the primary studies. Our meta-review also included many more primary studies than the Nieuwenhuijse et al. review [21].

Our study has several strengths. We used rigorous meta-review methodology, and applied stringent eligibility criteria to retrieve the highest quality of evidence available. We may not have identified all eligible studies based on the search strategy employed, and because registry studies and non-English language studies were excluded. Notably, there was little overlap of primary studies across HTAs, in part due to the fact that the HTAs differed in the span of years they covered. Primary studies varied in the consistency and completeness of information they reported so it was difficult to extract and summarize data. Given that included studies were published as early as 1986, some of the devices evaluated in included studies may no longer be used.

The Balliol Collaboration issued recommendations for improving the evidence base for surgical innovations [22]. However, in the current market approval process, uncontrolled clinical studies may suffice as evidence for device effectiveness and safety, so there is little incentive for manufacturers to undertake additional or rigorous studies of a more costly nature [23]. Furthermore, many medical devices are not marketed for long before they are replaced by newer versions, and therefore fail to undergo sufficient, long-term evaluation [24]. Given the rapid rate of new medical device development and marketing [25], and tensions between system-level funding policies and organizational purchasing decisions [26], future research should investigate how to generate, synthesize and share evidence on the safety and effectiveness of medical devices in a manner that balances innovation and safety. Others have suggested that improved regulation (pre-market) and professional society oversight (post-market) strategies are both needed to optimize patient safety [21].

Although users of health technologies are expected to use evidence to guide decisions about the use of medical devices, our study of knee arthroplasty implants—among the most commonly used implantable devices in major surgical procedures—suggests that little high-quality evidence actually exists. Our study raises serious questions about the nature of clinical evidence supporting the safety and effectiveness of implants used for knee arthroplasty

## Supporting Information

S1 Checklist PRISMA Checklist. (DOC)

S1 Table Medline search strategy. (DOCX)

S2 Table Web sites searched for HTAs. (DOCX)

S3 Table AMSTAR Scoring. (DOCX)

S4 Table Data extracted from primary studies and references. (DOCX)
