# Supplementary material for: Meta-Review of the Quantity and Quality of Evidence for Knee Arthroplasty Devices
Source: PLoS One. 2016 Oct 3;11(10):e0163032. doi: 10.1371/journal.pone.0163032 (PMC5047591; doi:10.1371/journal.pone.0163032)
Supplement: S1 Table — (DOCX) [file pone.0163032.s002.docx]

Supplemental Table 1. MEDLINE search strategy executed on January 14, 2015

1     Technology Assessment, Biomedical/ (5599)
2     arthroplasty, replacement, knee/ or knee prosthesis/ (15371)
3     1 and 2 (15)
4     limit 3 to (english language and yr="2005 - 2015") (8)
