# Supplementary material for: Meta-Review of the Quantity and Quality of Evidence for Knee Arthroplasty Devices
Source: PLoS One. 2016 Oct 3;11(10):e0163032. doi: 10.1371/journal.pone.0163032 (PMC5047591; doi:10.1371/journal.pone.0163032)
Supplement: S2 Table — (DOCX) [file pone.0163032.s003.docx]

Supplementary Table 2. Web sites searched for HTAs executed February 25, 2015

| **Organization** | Web site |
| --- | --- |
| National Institute for Clinical Excellence (UK) | https://www.nice.org.uk/guidance/published?type=ta |
| NHS National Institute for Health Research | http://www.nets.nihr.ac.uk/projects |
| Medicines and Healthcare Products Regulatory Agency | http://www.mhra.gov.uk/#page=DynamicListMedicines |
| University of York Centre for Reviews and Dissemination | http://www.crd.york.ac.uk/CRDWeb/ |
| Ontario Health Technology Assessment Committee | http://www.hqontario.ca/evidence/evidence-process/about-the-ontario-health-technology-advisory-committee |
| Canadian Association for Drugs and Technology in Health and Grey Matters (inventory of grey literature sources maintained by CADTH) | http://www.cadth.ca/  http://www.cadth.ca/en/resources/finding-evidence-is/grey-matters |
| Health Quality Council of Alberta | <http://www.hqca.ca/index.php?id=115> |
| Health Quality Ontario | <http://www.hqontario.ca/evidence/publications-and-ohtac-recommendations> |
| McGill University Health Centre Technology Assessment Unit | <http://www.mcgill.ca/tau/publications/> |
| Program for Assessment of Technology in Health | <http://www.path-hta.ca/Publications-Presentations/Publications/Reports.aspx> |
| EuroScan Secretariat. International Information Network on New and Emerging Health Technologies | <http://www.euroscan.org.uk/> |
| International Network of Agencies for Health Technology Assessment | <http://www.inahta.org/> |
| World Health Organization  Health Evidence Network  Medical Devices  Health Technology Assessments | <http://www.euro.who.int/en/what-we-do/data-and-evidence/health-evidence-network-hen/publications/by-keyword>  http://www.who.int/medical_devices/assessment/en/  http://www.who.int/medical_devices/en/ |
| Australian Government Department of Health and Ageing. Medical Services Advisory Committee Completed Assessments and Reviews | <http://www.msac.gov.au/internet/msac/publishing.nsf/Content/completed-assessments> |
| Queensland Government, Australia, Health Policy Advisory Committee on Technology | <http://www.health.qld.gov.au/healthpact/html/tech-evaluated.asp> |
| Royal Australian College of Surgeons. Australian Safety and Efficacy Register of new Interventional Procedures – Surgical | <http://www.surgeons.org/for-health-professionals/audits-and-surgical-research/asernip-s/systematic-reviews-and-technology-overviews/> |
| Australian Therapeutic Goods Administration | http://www.tga.gov.au/ |
| Health Technology Assessment, Australia | <http://www.health.gov.au/hta> |
| Institute of Technology Assessment | <http://www.oeaw.ac.at/ita/en/projects> |
| [Health Information and Quality Authority](http://www.hiqa.ie/about-us)**.** Health Technology Assessments | <http://www.hiqa.ie/healthcare/health-technology-assessment/assessments> |
| [National Health Service for Wales](http://www.attract.wales.nhs.uk/about.aspx) | <http://www.attract.wales.nhs.uk/> |
| [National Institute for Health and Clinical Excellence](http://www.nice.org.uk/aboutnice/) | <http://www.nice.org.uk/> |
| [National Institute of Health Research Horizon Scanning Centre](http://www.nhsc-healthhorizons.org.uk/about-us/) | <http://www.hsc.nihr.ac.uk/outputs/specialties/> |
| Agency for Healthcare Research and Quality Technology Assessments | <http://www.ahrq.gov/research/findings/ta/> |
| [Blue Cross and Blue Shield Association](http://www.bcbs.com/blueresources/tec/) Technology Evaluation Center | <http://www.bcbs.com/blueresources/tec/topic.html> |
| California Technology Assessment Forum | <http://www.ctaf.org/assessments> |
| [Centers for Medicare & Medicaid Services](http://www.cms.gov/About-CMS/About-CMS.html) Technology Assessments | <http://www.cms.gov/medicare-coverage-database/indexes/technology-assessments-index.aspx?TAId=85&bc=AAAQAAAAAAAA&> |
| [Institute for Clinical and Economic Review](http://www.icer-review.org/about/) | <http://www.icer-review.org/index.php/Table/Appraisals/> |
| United States Food and Drug Administration | http://www.fda.gov/MedicalDevices/ |
| Pan American Health Organization HTA Program | http://www.paho.org/hq/index.php?option=com_topics&view=article&id=131&Itemid=40823&lang=en |
| Health Technology Assessment International (HTAi) | http://www.htai.org/index.php?id=428 |
| EUnetHTA (Euro) | http://www.eunethta.eu/ |
