# Supplementary material for: Meta-Review of the Quantity and Quality of Evidence for Knee Arthroplasty Devices
Source: PLoS One. 2016 Oct 3;11(10):e0163032. doi: 10.1371/journal.pone.0163032 (PMC5047591; doi:10.1371/journal.pone.0163032)
Supplement: S3 Table — (DOCX) [file pone.0163032.s004.docx]

Supplemental Table 3. AMSTAR Scoring

| Study | ‘A priori’ design | Duplicate study selection and data extraction | Thorough literature search | Status of publication used as an inclusion criterion | List of studies (included and excluded) | Characteristics of the included studies | Scientific quality of included studies assessed and documented | Scientific quality of included used appropriately in formulating conclusion | Methods used to combine findings of studies were appropriate | Likelihood of publication bias assessed | Conflict of interest included | Total score (/11) |
| --- | --- | --- | --- | --- | --- | --- | --- | --- | --- | --- | --- | --- |
| Kane 2003 | Yes | Yes | No | No | Yes | Yes | Yes | Yes | Yes | No | No | 7 |
| CADTH 2013 | Yes | No | Yes | ? | No | Yes | Yes | No | No | No | No | 5 |
| Cerbo 2009 | Yes | No | Yes | No | Yes | Yes | Yes | Yes | No | No | Yes | 7 |
| Ethgen 2004 | Yes | No | Yes | No | Yes | Yes | No | No | No | No | No | 4 |
| Griffin 2005 | Yes | No | Yes | No | Yes | Yes | Yes | Yes | Yes | No | No | 7 |
| Hofstede 2015 | Yes | Yes | Yes | Yes | Yes | Yes | Yes | Yes | Yes | Yes | Yes | 11 |
| Kim 2014 | Yes | Yes | Yes | No | No | Yes | No | No | Yes | No | No | 5 |
| Medical Advisory Secretariat 2005 | Yes | No | Yes | Yes | No | Yes | Yes | No | Yes | No | No | 6 |
| Pabinger 2015 | Yes | Yes | Yes | Yes | Yes | Yes | No | Yes | Yes | Yes | Yes | 10 |
| Smith 2009 | Yes | Yes | Yes | Yes | No | Yes | No | No | Yes | Yes | No | 7 |
