# Supplementary material for: Meta-Review of the Quantity and Quality of Evidence for Knee Arthroplasty Devices
Source: PLoS One. 2016 Oct 3;11(10):e0163032. doi: 10.1371/journal.pone.0163032 (PMC5047591; doi:10.1371/journal.pone.0163032)
Supplement: S4 Table — (DOCX) [file pone.0163032.s005.docx]

Supplemental Table 4. Data extracted from primary studies and references

| Author | Year | Country | Focus | Study design | Study Objective | Device (model, company) | Patients (n) | Knees (n) | Follow-up (yrs) | Instrument or scale | Instrument score reported | Revision rate reported | Adverse events reported | Finding | Conflict of interest |
| --- | --- | --- | --- | --- | --- | --- | --- | --- | --- | --- | --- | --- | --- | --- | --- |
| Ackroyd | 2002 | UK | TKA, UKA | CC | UKA (medial) vs TKA | UKA St. Georg Sled, Waldemar Link; TKA Kinematic, Howmedica | 750 | 939 | mean 6 | Bristol | 1 | 1 | 1 | Range of motion improved significantly for UKA, no difference in revision rates | F |
| Adalberth | 2000 | Sweden | TKA | RCT | Cemented metal-backed vs all polyethylene | AGC Cemeted, Biomet | 34 | 40 | 2 | AKSS | 1 | 0 | 1 | Improve signficantly from baseline; no significant difference between groups | F |
| Aebli | 2004 | Switzerland | TKA | SC | Medial and lateral, cementless twin-bearing | LCS, Depuy | 91 | NR | mean 7.5 | AKSS, Insall | 1 | 1 | 1 | Radiolucent lines were not progressive | U |
| Aigner | 2004 | Austria | TKA | RCT | Deep-dish rotating platform vs mobile bearing | LCS, Depuy | 48 | 48 | 1 | HSS | 1 | 1 | 1 | Improve signficantly from baseline; no significant difference between groups | I |
| Alemparte | 2003 | Chile | TKA | SC | Cemented posterior cruciate-substituting design | AGC, Biomet | 76 (unclear how many deceased or lost to follow-up from this group) | 91 | 2 to 8 | AKSS (in person), variant of AKSS if over phone | 1 | 1 | 1 | No signficant difference | I |
| Ali | 2006 | UK | TKA | SC | Primary uncemented, rotating-platform, low-contact stress | Low Contact Stress, DePuy | 69 | 87 | 4-12, mean 6 years 8 months | AKSS | 1 | 1 | 1 | Improved from baseline, significance unclear | I |
| Ansari | 1997 | UK | UKA | SC | High tibial osteotomy vs. medial compartment replacement | St. Georg sledge prosthesis, Waldemar Link-Germany | 368 | 437 | 1-17, mean 4 | Bristol, VAS | 1 | 1 | 1 | Survival was 87% at 10 years with revision only as end-point to failure; method of fixation recommended | U |
| Ashraf | 2002 | UK | UKA | SC | Lateral UKA | St. Georg sledge prosthesis, Waldemar Link-Germany | 75 | 83 | 2-21, mean 9 | Bristol (before operation), OKA & WOMAC (after operation) | 1 | 1 | 1 | Improved from baseline, significance unclear | F |
| Asif | 2005 | Malaysia | TKA | SC | Midterm clinical and radiological results of TKA | Sigma Press Fit Condylar, DePuy, Warsaw IN | 44 | 79 | 4.5-6.4, mean 5.4 | AKSS, OKS | 1 | 1 | 1 | Improved from baseline, significance unclear | U |
| Attar | 2008 | UK | TKA | SC | Survival analysis of cemented TKA | Press Fit Condylar, Depuy, Leeds, UK | 160 | 207 | 0.3-16.9, mean 8.8 | Nottingham | 1 | 1 | 1 | Survival was 86% for worst-case scenario at 15 years, method of fixation recommended | I |
| Bachmeier | 2001 | Australia | TKA | SC | Comparison of validated instruments | NR | 108 | NR | mean 10 months | SF-36, WOMAC | 1 | 1 | 1 | Improved significantly from baseline using both instruments; WOMAC appears more responsive | F |
| Back | 2001 | UK | TKA | SC | Cumulative survival of cemented TKA over 5 years | Kinemax Knee System, Howmedica, Rutherford, UK | 310 | 364 | 4 years to 9 years 3 months, mean 5 years 8 months | AKSS | 1 | 1 | 1 | Survival was 99% at 5 years, method of fixation recommended | F |
| Bailey | 2014 | UK | TKA | RCT | Fixed-bearing vs. mobile-bearing cruciate retaining TKA | PFC sigma cruciate-retaining cobalt-chrome rotating platform (Depuy, Leeds, UK) or PFC sigma cruciate retaining titanium fixed-bearing platform (Depuy, Leeds, UK) | 331 (170 fixed, 161 rotating) | NR | 2 | AKSS, OKS, Patella, SF-12 | 1 | 1 | 0 | Improved from baseline; unclear significance; No significant difference between groups | U |
| Baker | 2007 | UK | TKA | RCT | Cumulative survival of cemented vs. cementless TKA over 15 years | press-fit condylar posterior cruciate ligament retaining knee replacement system, Johnson & Johnson Professional Inc., Raynham, Massachussetts | 214 | 269 | 5.8-16.8, mean 8.9 for cemented; 6.9-16.6, mean 8.7 in cementless | NR | 0 | 1 | 1 | Improved from baseline, unclear significance; No significant difference between groups | F |
| Baldwin | 1996 | USA | TKA | CC | Effect of bone quality on TKA outcomes | Intermedics Natural Knee (Intermedics Orthopedics Inc., Austin, Texas) | 300 | 364 (301 available for follow-up) | Mean 4, (range 2-65 months) | HSS | 1 | 1 | 1 | Improved from baseline, unclear significance; No significant difference between groups | U |
| Ballantyne | 2003 | UK | TKA | CC | Lateral release rates in press fit condylar prosthesis vs PFC Sigma prosthesis | PFC prosthesis modified by Sigma called PFC Sigma Knee, group 1; Sigma prosthesis, group 2 | 468, group 1; 369, group 2 | 542 group 1; 423 group2 | 6 months | AKSS | 1 | 1 | 1 | The use of the modified Sigma knee resulted in the reduction in the rate of lateral release; significance unclear | U |
| Bankes | 2003 | UK | TKA | SC | Metal-back tibia component among those with ideal and suboptimal alignment | Kinemax, Howmedica | 194 | 198 | mean 6.5 | AKSS | 1 | 1 | 1 | All groups improved from baseline, no significant difference across groups | F |
| Beard | 2007 | UK | TKA | SC | Assess mobile bearing implant complications | Total Meniscal Knee, Biomet | 172 | 172 | mean 2 | OKS | 1 | 1 | 1 | 59 (36%) reported a problem; no association with OKS score | U |
| Beard | 2007 | UK | TKA | RCT | Mobile vs. fixed | Total Meniscal Knee, Biomet (mobile); Anatomic Graduated Components, Biomet (fixed) | 33 | 66 | mean 3.7 | AKSS, OKS | 1 | 1 | 1 | No signficant differences between groups | U |
| Beaupre | 2007 | Canada | TKA | RCT | Cemented vs uncemented | Scorpio HA vs Scorpio Series 7000, Stryker | 81 | 81 | 5, (for 70 patients) | SF-36, WOMAC | 1 | 1 | 1 | No significant difference | F |
| Beaupre | 2001 | Canada | TKA | RCT | Mobilization by exercise-alone vs exercise + slider board vs exercise + continuous passive motion | NR | 93 | NR | 0.5 | SF-36, WOMAC | 1 | 1 | 1 | Significant improvement from baseline; no significant difference between groups | I |
| Berend | 2008 | USA | TKA | SC | Assess impact of body mass and smaller tibial implat on loosening | Anatomic Graduated Components, Biomet | 4393 | 6548 | 1 | AKSS | 1 | 1 | 1 | Higher body mass and smaller tibial component significantly associated with loosening | F |
| Berend | 2007 | USA | UKA | SC | Medial | Oxford, Biomet | 270 | 318 | mean 0.7 | AKSS | 1 | 1 | 1 | No significant difference by age or weight | F |
| Bert | 2000 | USA | TKA | SC | Long-term outcomes | Total Condylar design with either metal-backed modular tibial component or all-polyethylene tibial component (device and company name not provided) | 279 (5 lost to follow-up) | NR | 2 | SF-36 | 1 | 1 | 1 | Significantly improved from baseline | U |
| Bert | 2001 | USA | TKA | CC | Low-demand vs. medium/high-demand patients (based on activity level pre-op) | All-polyethylene tibial base or metal-backed cement-friendly tibial baseplate with a polyethylene insert (Wright Medical Technology, Arlington, Tennessee) Note: these were only for low-demand patients | 279 (185 low demand, 94 medium/high demand) | NR | 1 | Instrument name not specified but ‘Knee Scores’ and ‘Function Scores’ provided | 1 | 0 | 0 | Significantly improved from baseline; no significant difference between groups | U |
| Bertin | 2005 | USA | TKA | SC | Cruciate retaining | NexGen CR, Zimmer | 198 | 251 | mean 5.9, (for 165 patients) | AKSS, SF-12 | 1 | 1 | 1 | Significantly improved from baseline | F |
| Bhan | 2005 | India | TKA | RCT | Mobile vs. fixed | Low contact stress, Depuy (mobile); Insall Burstein II (fixed) | 32 | 64 | mean 6 | AKSS, OKS | 1 | 1 | 1 | Both groups improved significantly from baseline; no significant differences between groups | I |
| Bhan | 2003 | India | TKA | SC | Rotating platform LSC knee prostheses in rheumatoid and osteoarthritic knees with advanced stages of degeneration | LCS, Depuy, Warsaw, IN | 31 | 50 | mean 4.5 | AKSS | 1 | 1 | 1 | Significantly higher reoperation rate than other studies at 10%; method of fixation not recommended | I |
| Bietzel | 2013 | Germany | UKA | SC | Patellofemoral, Procedure for patellar stabilization | Journey, Smith & Nephew | 22 | NR | 2 | TL, VAS, WOMAC | 1 | 0 | 0 | Significantly improved from baseline | F |
| Birdsall | 1999 | UK | TKA | CC | Outcomes among patients aged 80 years+ | NR | 119 | NR | 1 | NHP | 1 | 0 | 0 | Significantly improved from baseline; significantly smaller improvement compared with younger cohort | I |
| Biswas | 2013 | USA | UKA | SC | Medial, Patients aged less than 55 yrs | Miller-Galante or Zimmer, Zimmer | 75 | 85 | mean 4 | KS, UCLA | 1 | 1 | 1 | Significantly improved from baseline | F |
| Borjesson | 2004 | Sweden | UKA Only | RCT | UKA (medial) vs HTO | UKA Robert Brigham, Johnson & Johnson | 18 | 18 | 5 | BOA, Borg | 1 | 1 | 1 | UKA - Improved from baseline | U |
| Boublik | 1993 | USA | TKA | SC | Cementless TKA in juvenile onset rheumatoid arthritis | PFC posterior cruciate sparing knee system, Johnson and Johnson, Braintree, MA | 14 | 22 | mean 3.9 | AKSS | 1 | 1 | 1 | Improved from baseline, significance unclear | U |
| Bozic | 2005 | USA | TKA | CC | Cruciate retaining vs stabilized | NexGen CR vs Nexgen LPS, Zimmer | 248 | 287 | mean 5.8 | HSS | 1 | 1 | 1 | No significant difference | F |
| Brander | 2003 | USA | TKA | SC | Cause of post-operative pain | NR | 116 | 149 | 1 | AKSS, BDI, HSS, McGill, PSS, STAI, VAS, WOMAC | 1 | 1 | 1 | Pain decreased significantly from baseline; influenced by preoperative pain, anxiety and depression | U |
| Brazier | 1999 | UK | TKA | SC | Compare rating instruments | NR | 109 | NR | 0.5 | EQ, HAQ, SF-36, WOMAC | 1 | 0 | 0 | Improved from baseline, unclear significance; WOMAC most responsive among rheumatology patients; HAQ and SF-36 equally responsive among osteoarthritis patients | I |
| Brown | 2001 | USA | TKA | SC | Assess component asymmetry in bilateral TKA patients | 2 implants from Howmedica and Zimmer (identical prosthesis type used in both knees) | 268 (246 at follow-up) | 536 | Min. 1, Mean 6.4 (range 1-2) | AKSS, HSS | 1 | 0 | 1 | Significant femoral component asymmetry for some patients; No significant difference between knees for asymmetric patients | U |
| Bruni | 2013 | Italy | UKA | SC | Medial, Minimal thickness all poly implant | Preservation, DePuy | 33 | NR | mean 8 | AKSS, OKS, TL, VAS, WOMAC | 1 | 1 | 1 | Significantly improved from baseline at 3 but not 8 years | U |
| Buechel | 2002 | USA | TKA | CC | Comparision of cementless posterior cruciate-retaining meniscal-bearing and cementless cruciate-sacrificing rotating-platform LCS TKA | New Jersey LCS TKA, Depuy, Warsaw, IN | 236 | 309 | mean 12.4 | NJOH | 1 | 1 | 1 | Improved from baseline, unclear significance; No significant difference between groups | F |
| Buechel | 2001 | USA | TKA | CC | Cemented and cementless comparision of mobile-bearing devices | New Jersey LCS TKA, Depuy, Warsaw, IN | 282 | 373 | mean 10.2 | NJOH | 1 | 1 | 1 | Improved from baseline, unclear significance; No significant difference between groups | U |
| Buechel | 1986 | USA | TKA, UKA (numbers NR) | SC | performance of new knee system | LCS Knee Replacement System, DePuy | 97 | 123 | mean 3.7 | Need to retrieve article | 1 | 1 | 1 | Improved from baseline, significance unclear | U |
| Bullens | 2001 | Netherlands | TKA | SC | Correlation of satisfaction with clinical outcome | Press Fit Condylar, Johnson & Johnson | 86 | 100 | mean 4.9 | AKSS, VAS, WOMAC | 1 | 1 | 1 | Improved from baseline; unclear significance. No correlation of VAS (patient-report) with AKS (physician-report), higher correlation with WOMAC (also patient-report) | I |
| Callaghan | 2005 | USA | TKA | SC | Report updated results of Callaghan 2000 study at minimum of 15 years | LCS rotating platform, Depuy, Warsaw, IN | 37 | 53 | minimum 15 | AKSS, HSS, WOMAC | 1 | 1 | 1 | Improved from baseline, unclear significance | F |
| Cameron | 1988 | Canada | TKA, UKA | CC | UKA vs TKA (each patient got one of each) | ICLH unicompartmental and total (Protek, Switzerland) or Tricon P unicompartmental and total (Richards Manufacturing Co., Memphis, TN) or Tricon M unicompartmental and total (Richards Manufacturing Co., Memphis, TN) | 20 | 20 | 2 | HSS | 1 | 1 | 1 | No significant difference between groups | U |
| Carlsson | 2005 | Sweden | TKA | RCT | cemented vs. cementless 5 year TKA results | PFC modular, posterior-cruciate-retaining prosthesis, Johnson and Johnson Orthopaedics, New Milton, UK | 96 | 120 | 5 | AKSS | 1 | 1 | 1 | Cementing of the tibial component offers more stable bone-implant contact for 5 years compared to uncemented fixation | U |
| Carr | 1993 | UK | TKA | SC | Results of medial UKA | Oxford Knee, Biomet, Warsaw, IN | 96 | 121 | 44.4 months | BOA | 1 | 1 | 1 | One third of patients improved; treatment appropriate in properly selected cases | U |
| Chiu | 2001 | Hong Kong | TKA | CC | Comparing results of mobile-bearing knee with fixed-bearing knee prosthesis | Low Contact Stress, DePuy; Anatomic Modular Knee, Depuy | 16 | 32 | min 1, mean 2 | AKSS, OKS | 1 | 1 | 1 | Significantly improved from baseline; no significant difference between treatments | U |
| Choy | 2011 | Korea | UKA | SC | Medial | Oxford, Biomet | 166 | 188 | mean 6.7 | HSS | 1 | 1 | 1 | Complications in 17 patients | I |
| Christensen | 1991 | Sweden | TKA, UKA | SC | UKA for gonathrosis | St. Georg-Schlitten, nonmetal-backed unicompartmental cemented prosthesis, Waldemar Link, Hamburg, Germany | 415 | 575 | NR; follow up evaluations at 3, 6 and 10 years | NR | 0 | 1 | 1 | Improved from baseline, unclear significance | U |
| Clark | 2001 | USA | TKA | RCT | Posterior stabilized vs cruciate retaining implants | AMK, DePuy, Johnson & Johnson | 108 | 108 | 2 | AKSS, SF-12, WOMAC | 1 | 0 | 0 | Improved from baseline; no significant differences between groups | U |
| Clayton | 2006 | UK | TKA | SC | Survival analysis of TKA | PFC Sigma TKA, Depuy, Johnson & Johnson | 212 | 180 | min 5, mean 61.7 months | AKSS | 1 | 1 | 1 | Improved from baseline, unclear significance; 97% survival at 5 years; method of fixation recommended | F |
| Clement | 2012 | UK | UKA | SC | Medial | Oxford, Biomet | 49 | 49 | mean 7.2 | OKS, VAS | 1 | 1 | 1 | 4 early failures (before 4 years) | U |
| Cloke | 2008 | UK | TKA | CC | Survival analysis of cemented and uncemented at 10 years | press-fit Kinemax Knee system, NR | NR; Unclear as some values are missing and breakdown of patients not provided | 89 | median 11 | NR | 0 | 1 | 1 | Cementless fixation had higher failure rate at 10 years, cemented fixation had acceptable 10 year cumulative survival | I |
| Cloutier | 2001 | Canada | TKA | SC | Outcomes when both cruciate ligaments retained | NR | 89 | 107 | mean 10 | AKSS | 1 | 1 | 1 | Improved from baseline, significance unclear | F |
| Cohen | 1997 | USA | TKA | CC | Unilateral vs simultaneous bilateral | Anatomic Modular Knee, DePuy | 186 | 172 | NR (minimum 6 months) | AKSS | 1 | 1 | 1 | No significant difference between groups | U |
| Confalonieri | 2004 | Italy | UKA | RCT | Medial, fixed vs mobile | Fixed Allegretto, Centerpulse; mobile AMC Unicondylar, Alphanorm | 40 | 40 | mean 5.7 | AKSS, GIUM | 1 | 1 | 1 | No significant difference between groups | U |
| Cool | 2006 | Belgium | UKA | SC | Medial, minimally invasive | Oxford, Biomet | 49 | 50 | mean 3.7 | AKSS | 1 | 1 | 1 | One failure and no complications | U |
| Dalury | 2008B | USA | TKA | SC | Midterm survival and radiographics results of TKA at 5 years | PFC knee system, Depuy Orthopaedics, Warsaw, IN | 986 | 1316 | mean 7.3 | AKSS | 1 | 1 | 1 | Significantly improved from baseline | F |
| Davis | 2007 | UK | TKA | SC | Assess to what degree polyethylene contributed to adverse events | Kinemax Plus, Stryker Howmedica, Rutherford, NJ | 19 | 19 | Retrospective; mean time to revision due to infection was 24.1 mo, revision secondary to aseptic causes was 27.4 mo | NR | 0 | 1 | 1 | With an original sample size of 1133 TKAs, premature failure is rare. Most failures were associated with surgeon error. | U |
| Dawson | 1998 | UK | TKA | SC | Compare rating instruments with novel instrument | NR | 85 | 85 | 0.5 | AKSS, HAQ, SF-36 | 1 | 0 | 0 | Performed well compared with existing instruments | I |
| Deshmukh | 2002 | UK | TKA | SC | Impact of body weight | Kinemax, Howmedica | 180 | NR | 1, (for 130 patients) | AKSS, NHP | 1 | 0 | 0 | Significantly improved from baseline; weight not associated with outcome | I |
| Diduch | 1997 | USA | TKA | SC | Patients aged 55 years or less | Cemented posterior stabilized cruciate-substituting systems | 84 | 103 | 8 | AKSS, HSS, TL | 1 | 1 | 1 | Improved from baseline, significance unclear | F |
| Dixon | 2005 | USA | TKA | SC | 15 year follow up of modular fixed-bearing posterior cruciate-retaining prostheses | PFC Total Knee Prosthesis, Johnson and Johnson Professional, Raynham, MA | 45 | 54 | mean 15.5 | AKSS | 1 | 1 | 1 | Improvement unclear as preop scores not taken; 15 year survivalship rate was 92.6% | F |
| Donnell | 1998 | UK | UKA | CC | Outcomes based on age (4 groups: 51-60, 61-70, 71-80, 81-90) | HLS Prosthesis (Tornier, St Ismer, France) | 221 | 221 | Min. 2, Avg. 3 | Guepar, HSS | 1 | 0 | 1 | Significantly improved from baseline; no difference between groups | U |
| Duffy | 2007B | USA | TKA | SC | 15 year survival analysis of femoral component failures in TKA | Hybrid TKA, Johnson and Johnson Press Fit Condylar, Raynham, MA | 57 | 65 | mean 15 | AKSS | 1 | 1 | 1 | Improved from baseline, unclear significance; Femoral component survivorship at 15 years was 72% | I |
| Duffy | 2007A | USA | TKA | SC | Long-term survival of primary cemented cruciate-retaining TKA in young patients with osteoarthritis | Press-Fit Condylar, Depuy, Warsaw, IN | 42 | 52 | median 12 | AKSS | 1 | 1 | 1 | Survival rate to revision 96% at 10 years and 85% at 15 years | I |
| Duffy | 1998 | USA | TKA | CC | Cemented vs uncemented | Press Fit Condylar, Johnson & Johnson | 102 | 108 | mean 10 | AKSS | 1 | 1 | 1 | Improved from baseline, no significant difference between groups; significantly more revisions in uncemented | U |
| Elke | 1995 | Switzerland | TKA | CC | Rheumatoid vs osteoarthritis | Porous Coated Anatomic, Howmedica | 394 | 524 | mean 4.2 (for 214 patients) | AKSS | 1 | 1 | 0 | Improved from baseline, significance unclear, no significant difference between groups | U |
| Emerson | 2008 | USA | UKA | SC | Determine limb alighment achieved in absence of ligament release and to investigate the interplay of failure mode, survivorship, and limb alignment | medial compartment Oxford phase-2 implant, Biomet, Warsaw, IN | 28 | 29 | mean 11.8 | AKSS | 1 | 1 | 1 | Survivorship at 10 years was 85%; mechanical limb alignment consistently through centre of knee | F |
| Emerson | 2004 | USA | UKA | CC | Compare the Oxford implant and the Replicci implant | Oxford knee implant, Bioment Europe, Dordrecht, The Netherlands; Replicci implant, Biomet, Warsaw, IN | 51 for Oxford; NR for Replicci | 55 for Oxford; 30 for Replicci | mean 10.2 for Oxford; mean 1.3 for Replicci | AKSS | 1 | 1 | 1 | Improved from baseline for both devices, significance unclear; No difference between devices | F |
| Emerson | 2002 | USA | UKA | CC | Medial, fixed vs mobile | Fixed Robert Brigham, DePuy; mobile Oxford, Biomet | 88 | 101 | mean 7.3 (for 78 patients) | AKSS | 1 | 1 | 1 | No significant difference between groups; reason for failure differed | U |
| Emerson | 2000 | USA | TKA | SC | Clinical results and survivorship of cemented AGC prostheses used for primary arthroplasty | Anatomic Graduated Components, Biomet, Warsaw, IN | 62 | 62 | mean 11.4 | AKSS | 1 | 1 | 1 | Improved from baseline, unclear significance; Survivorship at 11 years is 95% | F |
| Evanich | 1997 | USA | TKA | SC | Metal-backed patella | Natural Knee, Intermedics Orthopedics | 169 | 212 | mean 7.6 | HSS | 1 | 1 | 1 | Improved from baseline, significance unclear | U |
| Ewald | 1999 | USA | TKA | SC | Long term complications | Kinematic, Howmedica | 180 | 306 | NR | AKSS | 1 | 1 | 0 | Improved from baseline, significance unclear | U |
| Faris | 2008 | USA | TKA | SC | 13 year survivorship of AGC TKA | Anatomic Graduated Components, Biomet, Warsaw, IN | 131 | 201 | min 2 years, mean 7.9 | AKSS | 1 | 1 | 1 | Improved from baseline for both devices, significance unclear | I |
| Faris | 2006 | USA | TKA | CC | To compare two devices: the PCL retaining AGC and the posterior-stabilizing Legacy | PCL retaining Anatomic Graduated Components, Biomet, Warsaw, IN; posterior-stabilizing Legacy, Zimmer Warsaw, IN | 61 for AGC; 52 for Legacy | NR for both | mean 3.5 for AGC; mean 2.7 for Legacy | AKSS | 1 | 1 | 0 | No signficant difference between two methods of fixation | U |
| Faris | 2003 | USA | TKA | SC | To evaluate the clinical and radiographic results associated with the AGC all polyethylene tibial component | Anatomic Graduated Components, Biomet, Warsaw, IN | 405 | 536 | 1 | AKSS | 1 | 1 | 1 | Survival rate at 1 year is 90.04%, and at 10 years 68.11%; Low success rate among knees treated with this method of fixation are design sensitive | I |
| Fetzer | 2002 | USA | TKA | SC | To evaluate the immediate-term and long-term follow-up of PFC posterior cruciate-retaining TKA | Press-Fit Condylar, Johnson & Johnson, Raynham, MA | 56 | 78 | mean 10.5 | AKSS | 1 | 1 | 1 | Improved from baseline for both devices, significance unclear; survival was 100% at 10 years, and 93.3% at 12 years | F |
| Fitzgerald | 2004 | USA | TKA | SC | Physical function, pain | NR | 131 | 254 | 1 | SF-36 | 1 | 0 | 0 | Significantly improved from baseline | I |
| Forster | 2007 | Australia | UKA | CC | Medial and lateral, fixed vs mobile | Preservation, DePuy | 28 | 30 | 2 | AKSS, OKS | 1 | 1 | 1 | No significant difference between groups | U |
| Fortin | 1999 | Canada | TKA | CC | Pre-op vs. post-op physical function (2 pre-op cohorts: high vs. low physical function) | NR | 106 | 106 | 0.5 | SF-36, WOMAC | 1 | 1 | 1 | Significantly improved from baseline; high-function pre-op group had better post-op function | I |
| Geiger | 2008 | Germany | TKA | CC | Comparison of e.motion and PFC in a matched-pair analysis | e.motion, Aesculap; Press-fit condylar, Depuy | 60 | 66 | 2 | AKSS, FFbH-OA, OKS | 1 | 1 | 1 | No significant differences between preop and postop or between groups | U |
| Gill | 2001 | USA | TKA | SC | Long-term outcomes of cemented, PCL-retaining TKA | Total Condylar prosthesis (Howmedica) or Kinematic Condylar prosthesis with metal-backed tibial component (Howmedica) | 125 | 138 | Mean 16.8, (range 15.2-23.2) | AKSS | 1 | 1 | 1 | Significantly improved from baseline | U |
| Gill | 1999 | USA | TKA | SC | Assess outcomes of posterior cruciate retention implant | Total Condylar, Howmedica | 63 | 72 | mean 17.2 | AKSS | 1 | 1 | 1 | Significant improvement from baseline; 3 knees failed; 5 patients had complications; | U |
| Gioe | 2000 | USA | TKA | RCT | All-polyethylene vs metal backed tibial component | Press Fit Condylar, DePuy, Johnson & Johnson | 195 | 213 | mean 4.1 | AKSS, SF-36 | 1 | 1 | 0 | Improved from baseline, no significant difference between groups | U |
| Gleeson | 2004 | UK | UKA | RCT | Medial, fixed vs mobile | Fixed, St. Georg Sled, Waldemar; mobile Oxford, Biomet | 92 | 104 | mean 7 | Bristol, OKS | 1 | 1 | 1 | No significant difference between groups | U |
| Goldberg | 2004 | USA | TKA | SC | Cementless, with screw fixation | Miller-Galante, Zimmer | 99 | 124 | 14 | AKSS | 1 | 1 | 0 | Improved from baseline, unclear significance | I |
| Goodfellow | 1988 | UK | UKA | SC | Results of Oxford knee for unicompartmental arthritis | Oxford Knee; NR | NR | 98 | mean 36 months, median 33 months | BOA | 1 | 1 | 1 | Significantly improved from baseline | F |
| Griffin | 2007 | USA | TKA | SC | To determine outcomes of isolated tibial insert exchange in knees revised expressly for wear and osteolysis | Press-fit condylar, Depuy, J&J, Warsaw, IN | 68 | 68 | minimum 24 months, mean 44 months | AKSS | 1 | 1 | 1 | Improved from baseline, unclear significance | F |
| Griffin | 1998 | UK | TKA | CC | Obese vs non-obese patients | NR (cemented, posterior stabilized) | 56 | 73 | 10.6 | AKSS, HSS | 1 | 1 | 1 | Improved from baseline, no significant difference between groups | F |
| Griffiths | 1995 | Canada | TKA | SC | Comparing rating instruments | NR | 21 | NR | 0.5 | AIMS, HAQ, WOMAC | 1 | 0 | 0 | Significantly improved from baseline; WOMAC most responsive | U |
| Gupta | 2006 | USA | TKA | CC | A matched-pair study between PFC Sigma rotating-platform (RP) and PFC Sigma rotating-platform high flexion (RP-F) | Press-fit Condylar Sigma rotating platform high flexion, Depuy Orthopaedics, Inc, Warsaw, IN | 45 for PFC Sigma RP-F; 50 patients for Sigma RP | 50 for PFC Sigma RP-F; 50 for PFC Sigma RP | mean 12 months | AKSS | 1 | 1 | 1 | No significant differences between preop and postop or between groups, range of motion better in Sigma RP-F | U |
| Hansson | 2005 | Sweden | TKA | RCT | Mobile vs. fixed meniscal bearing | Rotaglide Total Knee System (Corin Medical Ltd., UK) or Nuffield Total Knee System (Corin Medical Ltd., UK) | 52 | 52 | 2 | HSS | 1 | 1 | 1 | No significant difference between groups | F |
| Hanusch | 2010 | UK | TKA | RCT | Fixed-bearing vs. rotating-platform | Press-fit condylar Sigma fixed-bearing system (PFC Sigma, Depuy) or Rotating-platform system (PFC Sigma RP, Depuy) | 120 (only 105 analyzed) | NR | Mean 13.4 months, (range 9.3 -283 mths) | AKSS, OKS | 1 | 1 | 1 | Improved from baseline, uncertain significance; No significant difference between groups | F |
| Hartford | 2001 | USA | TKA | SC | Results of low contact stress mobile bearing TKA | Low contact stress mobile bearing knee replacement, Depuy, Warsaw, IN | 66 | 92 | min 2 years | AKSS, WOMAC | 1 | 1 | 1 | Improvement unclear as preop scores not taken |  |
| Harwin | 1998 | USA | TKA | SC | Long terms results of implant with symmetrical femoral component | Kinemax, Howmedica | 326 | 356 | mean 5.1 | AKSS, HSS | 1 | 1 | 1 | Improved from baseline, significance unclear | U |
| Hasegawa | 2002 | Japan | TKA | SC | Factors influencing heterotopic ossification (HO) | NexGen CR (cemented) or NexGen LPS (uncemented), Zimmer | 140 | 221 | mean 3 | HSS | 1 | 0 | 1 | Significantly improved from baseline; HO identified in 9 patients (10 knees) | U |
| Hassaballa | 2003 | UK | TKA, UKA | CC | UKA vs patello-femoral vs TKA for kneeling ability | UKA St. Georg Sled, Waldemar Link; TKA Kinematic, Howmedica | 239 | 253 | up to 2 | OKS | 1 | 0 | 0 | Kneeling significantly improved in UKA patients | U |
| Healy | 2002 | USA | TKA | CC | Pre/post clinical pathway and all poly-ethylene tibial component | NR | 159 | 159 | 5 to 8 | AKSS, HSS | 1 | 1 | 1 | Mean hospital cost and LOS decreased significantly; no significant difference in function between groups | F |
| Heck | 1998 | USA | TKA | SC | Factors influencing outcome | NR | 291 | 330 | NR (minimum 2 years), (for 268 patients) | AKSS, SF-36, WOMAC | 1 | 1 | 1 | Significantly improved from baseline; hospital and surgeon volume, patient preoperative status, unilateral TKA positively associated with outcomes | I |
| Heller | 2009 | Israel | UKA | SC | Medial | Oxford, Biomet | 59 | 59 | mean 2.7 | AKSS, SF-12, WOMAC | 1 | 1 | 1 | Improved from baseline, 7 revisions | U |
| Henricson | 2006 | Sweden | TKA | RCT | Fixed polyethylene bearing vs. mobile polyethylene bearing | NexGen cruciate-retaining fixed-bearing cemented TKA (Zimmer) or MBK mobile-bearing cemented TKA (Zimmer) | 47 (45 at follow-up) | 52 (50 at follow-up) | 2 | AKSS | 1 | 0 | 1 | Improved from baseline, uncertain significance; No significant difference between groups | F |
| Himanen | 2007 | Finland | TKA | CC | To evaluate differences in the survival of moulded and modular AGC tibial components in patients with rheumatoid arthritis | Anatomic Graduated Components, Biomet, Warsaw, IN | 586 | 751 | mean 7.9 overall; 9.6 for moulded, 7 for modular | NR | 0 | 1 | 1 | No significant difference between survival of moulded and modular tibial components. | I |
| Hirsch | 1994 | USA | TKA | CC | To compare functional results in three groups: 1) those in which the posterior cruciate ligament was sacrificed, 2) those in which it was preserved, and 3) those in which the implant substituted for the resected posterior cruciate ligament | Press-fit condylar total knee prostheses, Johnson & Johnson, Raynham, MA (groups 1 and 2); Insall/Burstein II knee, Zimmer, Warsaw, IN (group 3) | NR | 77 for group 1; 80 for group 2; 85 for group 3 | minimum 2 years; mean 2.7 for group 1, mean 2.7 for group 2, mean 2.6 for group 3 | AKSS | 1 | 1 | 1 | No significant differences between preop and postop or between groups, range of motion better in group 3 (posterior cruciate ligament-substituting device) | U |
| Hooper | 2012 | New Zealand | UKA | SC | Medial Cementless | Oxford, Biomet | 185 | 196 | 2 | HAAS, OKS | 1 | 0 | 0 | Significantly improved from baseline | I |
| Hsu | 1998 | Taiwan | TKA | SC | Hybrid technique: uncemented femoral component and cemented tibial and patellar components | Miller Galante I Prosthesis (Zimmer, Warsaw, IN) | 113 | 140 | Mean 4.8 (range 3.2-6.6) | HSS | 1 | 1 | 1 | Significantly improved from baseline | U |
| Huang | 2005 | Taiwan | TKA | SC | Classification of types of patellar polyethylene failure | New Jersey Low contact stress knee system, Depuy, Warsaw, IN | NR | 598 | range 5-12 years | NR | 0 | 1 | 1 | Two factors were identified as reason for patellar polyethylene failure: misaligned contact surfaces and blockage of rotating mobility | I |
| Huang | 2003 | Taiwan | TKA | SC | To assess survivorship and long-term results of mobile-bearing TKA | Low contact stress mobile bearing knee replacement, Depuy, Warsaw, IN | 406 | 495 | mean 12 years | AKSS | 1 | 1 | 1 | Overall surviorship at 15 years was 88.1% | I |
| Hyldahl | 2005B | Sweden | TKA | RCT | All-polyethylene vs metal backed tibial component | Anatomic Graduated Components, Biomet, Warsaw, IN | 38 | 40 | 2 | HSS | 1 | 1 | 1 | Improved from baseline, unclear significance; No significant differences between groups | I |
| Hyldahl | 2005A | Sweden | TKA | RCT | All-polyethylene vs metal backed tibial component | Anatomic Graduated Components, Biomet, Warsaw, IN | 40 | 40 | 2 | HSS | 1 | 1 | 1 | Improved from baseline, unclear significance; No significant differences between groups | I |
| Ikejiani | 2000 | Canada | TKA | CC | Resurfacing vs non-resurfacing of the patella | Genesis, Smith & Nephew | 185 | 185 | 2 | HSS | 1 | 1 | 1 | No significant difference between groups | U |
| Indelli | 2002 | Italy | TKA | SC | Assess outcomes at a minimum of five years | Insall-Burnstein Posterior Stabilized, Zimmer | 85 | 92 | 7.5 | AKSS | 1 | 1 | 1 | Improved from baseline, significance unclear | I |
| Ivarsson | 1991 | Sweden | UKA Only | CC | UKA (medial) vs HTO | UKA Oxford Knee, Biomet and Porous Coated Anatomic, Howmedica | 10 | 10 | 1 | Lysholm, VAS | 1 | 0 | 0 | UKA - Improved from baseline | U |
| Jacobs | 2011 | Netherlands | TKA | RCT | Fixed vs. mobile bearing | BalanSys (Mathys Medical Ltd. Bettlach, Switzerland) | 92 | NR | 1 | AKSS | 1 | 1 | 1 | No significant difference between groups for active knee flexion; Fixed group had significantly better stair climbing score | U |
| Jahromi | 2004 | Australia | UKA | SC | Assessment of patient outcomes following UKA with mini-incision | Oxford UKA, Biomet Inc, Warsaw, IN | 150 | 183 | minimum 12 months | Grimby, OKS, VAS | 1 | 0 | 0 | Improved from baseline, unclear significance | U |
| Jain | 2011 | USA | UKA | SC | Medial, Novel implant design features | Preservation, DePuy | 71 | 72 | 2, (for 57 patients) | AKSS, WOMAC | 1 | 1 | 1 | Significantly improved from baseline | I |
| Jeer | 2004 | Australia | UKA | SC | Analysis of LSC UKA failures at 5 years | Low contact stress UKA system, Depuy, Leeds, UK | NR | 58 | 5.9 | OKS | 1 | 1 | 1 | Significantly improved from baseline; survival at 5 years was 89.7% | U |
| Jenny | 1998 | France | TKA | CC | Cruciate-retaining vs cruciate-replacing | Search, Aesculap | 125 | 125 | 2 to 3 | AKSS | 1 | 1 | 1 | Improved from baseline, significance unclear; no significant difference between groups | U |
| Jones | 2003 | Canada | TKA | SC | Factors associated with improvement | NR | 276 | 276 | 0.5 | SF-36, WOMAC | 1 | 0 | 0 | Improved from baseline, unclear significance, influenced by preoperative function | I |
| Jones | 2000 | Canada | TKA | SC | Quality of life outcomes | NR | 276 | 276 | 0.5 | SF-36, WOMAC | 1 | 1 | 1 | Significantly improved from baseline | I |
| Jones | 2001 | Canada | TKA | CC | Outcomes among patients aged 80 years and older vs 79 years and younger | NR | 257 | 257 | 0.5 | SF-36, WOMAC | 1 | 1 | 1 | Significantly improved from baseline; no significant difference between groups | I |
| Jordan | 1997 | USA | TKA | SC | Assess cementless, mobile bearing implant | Primary Low Contact Stress, DePuy | 375 | 472 | mean 4.7, (for 410 patients) | AKSS | 1 | 1 | 1 | Improved from baseline, significance unclear | U |
| Joshi | 2003 | USA | TKA | SC | Outcomes for patients aged 80 and older | Total Condylar, Kinematic, Howemdica; Anatomic Graduated Component, Gill Precision Anatomic Component, Biomet | 90 | 110 | mean 7.9, (for 25 patients at final follow-up) | AKSS | 1 | 1 | 1 | Significantly improved from baseline | I |
| Kageyama | 1998 | Japan | TKA | CC | Pre-op vs post-op conditions (4 groups based on # of arthroplasties) | NR | 73 | 110 (Note: had to add up these numbers myself) | 2 | Face, mHAQ | 1 | 0 | 1 | Face Scale scores significantly improved from baseline | U |
| Kasodekar | 2006 | Singapore | UKA | SC | Evaluate the clinical outcome of open standard UKA and establish influence of radiological alighment on survivorship | Press fit condylar unicompartmental knee system, Depuy, Leeds, UK | 15 | 18 | 4 to 8 | AKSS | 1 | 1 | 0 | Significantly improved from baseline; survival at 4 years was 91.7% | U |
| Katz | 1994 | USA | TKA | SC | Pre-op vs. post-op functional status | NR | 172 | 172 | 0.5 | IADL | 1 | 0 | 1 | Improved from baseline, unclear significance | I |
| Keating | 2002 | USA | TKA | SC | Evaluate and determine the mechanism and etiology of failure of components that failedin long-term follow up of AGC TKA | nonmodular metal-backed tibia components anatomic graduated component, Biomet Inc, Warsaw, IN | 3054 | 4583 | Up to 17 years, mean unclear | NR | 0 | 1 | 1 | Method of fixation proved to have minimal wear and excellent logevity with time with no revisions between 10-15 years | U |
| Keblish | 2004 | France and USA | UKA | SC | Results of LCS mobile-bearing UKA in patients with medial or lateral component arthritis | Low contact stress unicompartmental prosthesis, Johnson & Johnson/Depuy, Warsaw, IN | 100 | 147 | mean 11 | NJOH | 1 | 1 | 1 | Improved from baseline, unclear significance; Survivorship at 11 years is 82% | F |
| Keblish | 1994 | USA | TKA | CC | Comparision between resurfaced vs. not resurfaced patella in patients with bilateral TKA | Low contact stress, Depuy, Warsaw, IN | 52 | 104 | 5.24 | HSS | 1 | 1 | 1 | No signficant difference between two methods of fixation | I |
| Kempshall | 2009 | UK | TKA | SC | Compare results of surgery performed at NHS Teatment Centre to those published at other centres | Kinemax total knee system, Stryker, Howmedical, Rutherford, NJ | 175 | 206 | mean 2 | AKSS | 1 | 1 | 1 | Results of patients treated at NHS Treatment Centre significantly worse than those at other centres | I |
| Kim | 2009b | Korea | TKA | RCT | Fixed vs. mobile bearing | Anatomic Modular Knee (AMK, Depuy) for fixed-bearing or Low Contact Stress (LCS, Depuy) for mobile | 61 | 122 | Mean 10. 8 (range 10-12) | AKSS, HSS | 1 | 1 | 1 | Improved from baseline, unclear significance; No significant difference between groups | I |
| Kim | 2009a | Korea | TKA | RCT | Fixed vs. mobile bearing | Medial Pivot fixed-bearing (Wright Medical) or PFC Sigma mobile-bearing (Depuy) | 92 | 184 | Mean 2.6 (range 2-3) | AKSS, HSS | 1 | 1 | 1 | Significantly worse scores for Medial Pivot fixed-bearing prosthesis | I |
| Kim | 2010 | Korea | TKA | RCT | Fixed vs. mobile bearing | Mobile-bearing prosthesis: e.motion-FP (B.Braun-Aesculap, Tuttlingen, Germany) or Fixed-bearing prosthesis: Genesis II (Smith and Nephew, Memphis, USA) | 66 | 132 | 2 | AKSS, WOMAC | 1 | 0 | 0 | Improved significantly from baseline; no significant difference between groups | U |
| Kim | 2007 | Korea | TKA | RCT | Comparison between fixed-bearing and mobile-bearing TKR - one in each per patient (bilateral) | Fixed-bearing anatomic modular knee total knee prosthesis, Depuy, Warsaw, IN; low contact stress mobile-bearing rotating-platform total knee prosthesis, Depuy | 146 | 292 | mean 13.2 | AKSS, HSS | 1 | 1 | 1 | Improved from baseline, unclear significance; No signficant difference between two methods of fixation | I |
| Kim | 2004 | Korea | TKA | SC | Anterior-posterior glide vs rotating platform, each patient received one of each | Anterior-Posterior Glide, Low Contact Stress, DePuy | 190 | 380 | mean 6.4 | AKSS, HSS | 1 | 0 | 0 | Improved from baseline, no significant difference across knees | I |
| Kim | 2001 | Korea | TKA | RCT | Fixed vs. mobile bearing | Anatomic Modular Knee (AMK, Depuy) for fixed-bearing or Low Contact Stress (LCS, Depuy) for mobile) | 116 | 232 | Mean 7.4, (range 6-8) | AKSS, HSS | 1 | 1 | 1 | Improved from baseline, unclear significance; No significant difference between groups | U |
| Kim | 2007 | Korea | TKA | RCT | Fixed vs. mobile bearing | Fixed or mobile PFC Sigma (Depuy) | 174 | 348 | Mean 5.6 (range 5.2 – 6.1) | AKSS, HSS | 1 | 1 | 1 | No significant difference between groups | I |
| Kim | 2007 | Korea | UKA | SC | Medial, minimally invasive | Oxford, Biomet | 68 | 95 | minimum 3 | AKSS | 1 | 0 | 1 | Significantly improved from baseline | I |
| Konig | 2000 | Germany | TKA | SC | Uncemented femoral component | Press Fit Condylar, Johnson & Johnson | 225 | 253 | mean 5.3 | AKSS | 1 | 0 | 1 | Significantly improved from baseline | F |
| Kort | 2007 | Netherlands | UKA | SC | Medial, minimally invasive | Oxford, Biomet | 132 | 154 | 2 to 7 | AKSS, SF-36, WOMAC | 1 | 1 | 1 | Improved from baseline, 17 revisions | I |
| Kort | 2007 | Netherlands | UKA | SC | Evaluate midterm results of Oxford phase III UKR in patients 60 or younger | Oxford phase III unicompartmental knee replacement; NR | 43 | 46 | minimum 2, maximum 6 | AKSS, SF-36, WOMAC | 1 | 1 | 1 | Improved from baseline, unclear significance | U |
| Kramers-de Quervain | 2005 | Switzerland | TKA | SC | assess performance of new tibial component | LCS, DePuy | 218 | 230 | mean 15.8 months | NR | 0 | 1 | 1 | 24 knees (10.4%) needed revision which is higher than other implant systems | I |
| Kreibich | 1996 | Canada | TKA | SC | Compare rating instruments | NR | 68 | NR | 0.5 | AKSS, SF-36, WOMAC | 1 | 0 | 0 | Significantly improved from baseline; WOMAC and SF-36 most responsive | U |
| Krüger (first author is actually Parsch) | 2009 | Germany | TKA | SC | Results of nonmodular fixed-bearing TKA | Pressfit condylar, Depuy Orthopaedics, Inc. | 58 | 58 | mean 13 | AKSS, FFbH-OA, OKS, VAS | 1 | 1 | 1 | Significantly improved from baseline (Knee Society scores), but not for the Knee Society function scores; Other assessments, significance unclear; Survival at 10 and 14 years is 97%. | U |
| Lampe | 2011 | Germany | TKA | RCT | Fixed vs. mobile bearing | Fixed-bearing implant: Columbus CR (B.Braun Aesculap) or Mobile-bearing implant: Columbus RP (B.Braun Aesculap) | 100 | 100 | 1 | AKSS, OKS | 1 | 1 | 1 | No significant difference between groups | F |
| Langdown | 2005 | Sweden | UKA | CC | Assess outcome of UKA using Oxford prosthesis for end-stage focal spontaneous osteonecrosis of the knee | Oxford Media UKA, Biomet-Merck, Bridgend, UK | 26 control; 27 osteonecrosis | 28 control; 28 osteonecrosis | Mean 4.8 control; mean 5.2 osteonecrosis | OKS | 1 | 1 | 1 | Improved from baseline, significance unclear; no significant difference between groups | I |
| Larson | 2001 | USA | TKA | CC | One vs three peg patellar fixation | Insall-Burnstein, Zimmer | 82 | 118 | mean 5.4 | HSS | 1 | 1 | 1 | No significant postoperative differences between groups | U |
| Laurencin | 1991 | USA | TKA, UKA | SC | Each patient received one UKA and one TKA knee | UKA Robert Brigham and Unicondylar, Johnson & Johnson; TKA Kinematic, Howmedica or Press-fit Condylar or Duopatellar metal backed system, Johnson & Johnson | 48 | 48 | mean 6.75 | Asked patients by phone to report pain, stability, “feel”, and ability to use stairs | 0 | 1 | 1 | No significant difference | U |
| Lavernia | 1997 | USA | TKA | SC | Quality of life | Duracon, Howmedica | 100 | 127 | 1, (for 52 patients) | QWB | 1 | 0 | 0 | No significant difference | F |
| Leonard | 2003 | UK | TKA | SC | Comparision patients with simultaneous bilateral TKAs (much of unilateral cohort data missing, so excluded here) | Press Fit Condylar, DePuy, Johnson & Johnson company, Leeds, England | 57 bilateral | 114 bilateral | mean 66 months bilateral | AKSS | 1 | 1 | 1 | Significance unclear between two groups; No preop AKS scores taken; Survivorship at 7 years is 97% for bilateral | F |
| Li | 2006 | Australia | UKA | RCT | Medial, fixed vs mobile | Fixed Miller Galante, Zimmer; mobile Oxford, Biomet | 48 | 56 | 2 | AKSS, SF-36, WOMAC | 1 | 1 | 1 | Improve significantly from baseline; no significant difference between groups | U |
| Liddle | 2013 | UK | UKA | SC | Medial, Cementless | Oxford, Biomet | 881 | 1,000 | 1, (for 959 knees) | OKS | 1 | 1 | 1 | No significant difference from baseline | F |
| Lim | 2012 | Korea | UKA | SC | Medial | Oxford, Biomet | 320 | 400 | mean 5.2 | AKSS, OKS | 1 | 1 | 1 | Significantly improved from baseline; 14 revisions | I |
| Lingard | 2001 | USA | TKA | SC | Analyze patient recall of postoperative status | NR | 770 | 770 | 3 mo | AKSS, SF-36, WOMAC | 1 | 1 | 1 | Patients' recall of preoperative pain and functional status 3 months post-op only demonstrated moderate agreement with what patients had reported prospectively | F |
| Lingard | 2001 | USA | TKA | SC | Compare rating instruments | Kinemax, Howmedica | 660 | 660 | 0.25 | AKSS, SF-36, WOMAC | 1 | 0 | 0 | Improved from baseline, unclear significance; WOMAC and SF-36 more responsive than AKS | F |
| Lisowski | 2011 | Netherlands | UKA | SC | Medial, minimally invasive | Oxford, Biomet | 216 | 244 | median 4.2 | AKSS, OKS, VAS, WOMAC | 1 | 1 | 1 | Improved from baseline, 9 revisions | I |
| Lisowski | 2004 | Netherlands | UKA | SC | Evaluate UKA for anteromedial osteoarthritis | Oxford phase III unicompartmental knee replacement; NR | 28 | 30 | minmum 2 years, mean 2.54 | AKSS | 1 | 1 | 1 | Significantly improved from baseline | U |
| Liu | 1998 | Taiwan | TKA | CC | Simultaneous vs consecutive bilateral TKA | Porous Coated Anatomic, Howmedica; Miller-Galante, Zimmer; Osteonics, Omnifit; Whiteside Ortholoc, Dow Corning | 88 | 176 | 2.6 | HSS | 1 | 0 | 1 | No significant postoperative differences between groups | U |
| Lizaur-Utrilla | 2012 | Spain | TKA | RCT | Fixed vs. mobile bearing | Trekking Mobile-bearing (Samo, Bologna, Italy) or Multigen Plus Fixed-bearing (Lima, Udine, Italy) | 119 | 119 | Mean 30.3 mths (fixed group); mean 30.4 mths (mobile group) | AKSS, SF-12, VAS, WOMAC | 1 | 1 | 1 | Improved from baseline, unclear significance; Mobile group had significantly greater knee flexion at 3 and 6 months (but no sig. difference between groups at 2 years) | I |
| Lombardi | 2009 | USA | TKA, UKA | CC | Mobile, medial, minimally invasive UKA vs cruciate retaining TKA | UKA Oxford, Biomet; TKA Vanguard, Biomet | 206 | 230 | mean 2.6 | OKS | 1 | 1 | 1 | No significant difference between groups | F |
| Lombardi | 2001 | USA | TKA | CC | Pathology-based algorithm for posterior cruciate retaining vs stabilized | Maxim Complete, Biomet | 240 | 351 | mean 6.2 | AKSS, HSS | 1 | 1 | 1 | Significantly improved from baseline; no significant difference between groups | U |
| Lonner | 2001 | USA | TKA | CC | To determine if tibial stem design affects bone density in the longterm | Miller-Galante TKA, Zimmer, Warsaw, IN; Press-fit Condylar TKA, Johnson & Johnson, Rayham, MA | 12, 6 per device | 12, 6 per device | mean 95 months in MG; mean 94 months in PFC | AKSS | 1 | 0 | 0 | Improved from baseline, significance unclear; Bone densities under medial and lateral plateaus significantly reduced in PFC group compared to MG group | I |
| Luscombe | 2007 | UK | UKA | SC | Medial, minimally invasive | Oxford, Biomet | 68 | 78 | 2, (for 60 patients) | AKSS, OKS | 1 | 1 | 1 | Improved from baseline, 4 revisions | U |
| Lyback | 2004 | Finland | TKA | SC | To elucidate whether patellar resurfacing would reduce pain after TKA in juvenile rheumatoid arthritis | Anatomically Graduated Component, Biomet, Warsaw, IN | 48 | 71 | mean 7.3 | NR | 0 | 1 | 1 | Patient satisfaction was higher in those with replaced patella, though significance unclear; authors recommend resurfacing | U |
| Lyback | 2000 | Finland | TKA | SC | Survivorship of knee replacement in patients with juvenile chronic arthritis | Anatomically Graduated Component, Biomet, Warsaw, IN | 48 | 73 | 3 to 13 years | NR | 0 | 1 | 1 | Survival at 5 years is 99% | I |
| Mackinnon | 1988 | UK | UKA | SC | Review of the St. Georg sledge arthroplasty | St. Georg sledge prosthesis (designed by Buchholz at the St Georg Hospital in Hamburg) | 100 | 115 | mean 57 months | Bristol | 1 | 1 | 1 | Improved from baseline, significance unclear | U |
| March | 2004 | Australia | TKA | SC | Unilateral vs bilateral TKR | NR | 207 | 278 | 1 | SF-36, WOMAC | 1 | 0 | 0 | Improved from baseline, no significant difference across groups | I |
| Martin | 1997 | USA | TKA | SC | Outcomes at a minimum five year follow-up | Press Fit Condylar, Johnson & Johnson | 231 | 306 | mean 6.5 | AKSS | 1 | 1 | 1 | Improved from baseline, significance unclear | U |
| Maruyama | 2004 | Japan | TKA | RCT | RCT of posterior cruciate-retaining and posterior stabilized TKAs in patients with bilateral TKAs for osteoarthritis | posterior cruciate-retaining and posterior stabilized TKAs, both by Depuy, Johnson & Johnson, Warsaw, IN | 20 | 40 | mean 31.7 months | AKSS | 1 | 0 | 1 | Significantly improved from baseline; No signficant difference between two methods of fixation | I |
| Matharu | 2012 | UK | UKA | SC | Medial, Influence of age | Oxford, Biomet | 392 | 459 | mean 4.4 | OKS | 1 | 1 | 1 | Significantly improved from baseline, age not associated with outcomes | I |
| Matsueda | 2000 | Japan | TKA | CC | Medial parapatellar vs subvastus approach | Genesis, Smith & Nephew | 291 | 336 | 0.5 | AKSS | 1 | 1 | 1 | No significant difference between groups in knee score; patella tracked centrally in significantly more, and required significantly fewer release procedures in the subvastus group | U |
| Mayman | 2003 | Canada | TKA | RCT | Resurfaced vs non-resurfaced patella | Anatomic Medullary Knee, DePuy | 100 | NR | 8 to 10 | AKSS | 1 | 1 | 1 | AKS scores did not differ significantly between groups; walking and climbing pain significantly less in resurfaced group | I |
| McCaskie | 1998 | UK | TKA | RCT | Cemented vs uncemented | Condylar PFC, Johnson & Johnson | 113; 67 cemented, 47 uncemented | 139; 81 cemented, 58 uncemented | 5 | Nottingham | 1 | 1 | 1 | No significant difference | F |
| McGuigan | 1995 | USA | TKA | SC | Long-term follow-up | AGC (Biomet) IB-II (Zimmer, Warsaw, IN) | NR | NR | 2 | SF-36 | 1 | 0 | 1 | Improved significantly from baseline (except for patient’s health perception) | U |
| Meding | 2003 | USA | TKA | CC | Diabetic vs non-diabetic patients, with cefuroxime in the cement | Anatomic Graduated Component, Biomet | 3,519 | 5,220 | 4.5 | AKSS | 1 | 1 | 1 | Post-operative knee scores significantly higher in diabetic group but function scores lower | F |
| Meding | 2001 | USA | TKA | SC | Influence of severity of osteoarthritis | Anatomic Graduated Component, Biomet or Insall-Burstein, Zimmer | 1,888 | 2,759 | mean 2.5 | AKSS | 1 | 0 | 0 | Improved from baseline, significance unclear; no significant difference in outcomes between groups | I |
| Meding | 2001 | USA | TKA | CC | Thicker (6.4mm) vs. thinner (4.4) tibila polyethylene in TKA | Anatomic Graduated Components, Biomet, Warsaw, IN | 116 (bilateral) | 132; 116 thicker, 116 thinner | mean 10.7 | AKSS | 1 | 1 | 1 | No signficant difference between two methods of fixation; Preop results not declared | U |
| Mikulak | 2001 | USA | TKA | SC | To determine the factors that predispose or contribute to aseptic loosening and osteolysis | Press-fit condylar total knee prosthesis with posterior-cruciate-substituting-design, Johnson and Johnson Orthopaedics, Raynham, MA | 15 | 16 | mean 56 months | AKSS | 1 | 1 | 1 | Improved from baseline, significance unclear | F |
| Miner | 2003 | USA | TKA | SC | Correlation of range of motion with function | Kinemax (Stryker Howmedica) | 684 | 684 | 1 | WOMAC | 1 | 0 | 0 | Range of motion and function both improved but were not correlated | F |
| Miyasaka | 1997 | USA | TKA | SC | Long term follow-up ligament balancing approach for valgus knee deformity with | Total Condylar, Johnson & Johnson or Howmedica | 46 | 60 | mean 14 | AKSS | 1 | 1 | 1 | Improved from baseline, significance unclear; 24% instability | U |
| Mokris | 1997 | USA | TKA | CC | Cemented vs uncemented femoral component | Genesis Total Knee, Smith & Nephew | 90 | 105 | mean 4.25 | AKSS | 1 | 1 | 1 | Significantly improved from baseline, no significant difference between groups | U |
| Mont | 1999 | USA | TKA | SC | Minimum five year results, particular emphasis on patellofemoral complications | Duracon Total Knee, Howmedica | 101 | 118 | mean 5.4 | AKSS | 1 | 1 | 1 | Improved from baseline, significance unclear; no patellofemoral compliations | U |
| Morgan | 2008 | UK | TKA | CC | To examine the association between postop coronal alignment in TKA in 1) neutral, 2) valgus and 3) varus groups | Kinemax knee arthroplasty, NR | 153 | 197 | mean 9 | NR | 0 | 1 | 0 | No significant association between postop radiographic coronal knee alignment and revision surgery; No significant difference between groups |  |
| Moskal | 1998 | USA | TKA | SC | Long-term follow-up | Constrained Condylar Knee (Zimmer Inc., Warsaw, Indiana). PCL-retaining designs or PCL-subsituting designs or posterior-stabilized, semi-constrained designs. 490 of 589 primary TKAs used hybrid fixation; 99 had all components cemented | 514 | 646 (589 primary, 57 revision) | Mean 4.3, (range 2-8) | HSS | 1 | 1 | 1 | Improved from baseline, unclear significance | U |
| Mullaji | 2011 | India | UKA | SC | Medial, minimally invasive | Oxford, Biomet | 109 | 122 | NR | NR | 0 | 0 | 1 | Limb alignment improved | F |
| Munro | 2010 | New Zealand | TKA | RCT | Fixed vs. mobile bearing | PFC Sigma fixed-platform (Depuy) or PFC Sigma rotating-platform (Depuy) | 41 | 48 | 2 | AKSS, OKS, SF-12, VAS, WOMAC | 1 | 1 | 1 | No significant difference between groups | F |
| Munzinger | 2000 | Switzerland | TKA | SC | LCS TKA with metal backed rotating bearing patella components | Low contact stress, Depuy, Leeds, UK | 105 | 105 | minimum 2 years, mean 4.2 | HSS (modified) | 1 | 1 | 1 | Improved from baseline, significance unclear | U |
| Murray | 1998B | UK | UKA | SC | 10 year survival of knees with anteromedial osteoarthritis and normal ACLs | Oxford Meniscal prosthesis, Biomet Ltd., Bridgend, UK | 113 | 143 | mean 7.6 | NR | 0 | 1 | 1 | Survival rate at 10 years is 98% |  |
| Murray | 1998A | UK | TKA | CC | comparison of survival based on pain rather than revision rate | AGC, Biomet; IB2, Zimmer; Nuffield Knee, Corin Medical | NR | 1,429 | 3.5 | NR | 0 | 1 | 1 | Significantly more patients with Nuffeld knee developed moderate pain | F |
| Nafei | 1992 | Denmark | TKA | CC | Comparision between Kinemax (cemented, hybrid and cementless) and Insall-Burstein prosthesis (control, all cemented) | Kinemax prosthesis, Howmedical, UK; cemented Insall-Burstein prosthesis | 72 Kinemax; 46 Insall-Burstein | 75 Kinemax; 50 Insall-Burstein | Kinemax mean 14 mo, NR Insall-Burstein | HSS | 1 | 1 | 0 | Improved from baseline, unclear significance; Preop to postop HSS score gain significantly greater in Kinemax cemented group; No comparisions to control group made | I |
| Nerhus | 2012 | Norway | UKA | SC | Medial, Patient-reported outcomes | Oxford, Biomet | 96 | 99 | 2 | KOOS, OKS, UCLA | 1 | 1 | 1 | No significant difference from baseline | I |
| Newman | 2009 | UK | TKA, UKA | RCT | UKA (medial, lateral) vs TKA | UKA St. Georg Sled, Waldemar Link; TKA Kinematic, Howmedica | 9 for UKA; 13 for TKA | 13 for UKA, 17 for TKA | 15 | Bristol | 1 | 1 | 1 | No significant difference in Bristol score; range of motion improved significantly for UKA | F |
| Nielsen | 1992 | Denmark | TKA | CC | Results and survivorship of uncemented UKAs in osteoarthritis vs. rheumatoid arthritis | Anatomic Graduated Components, Biomet, Warsaw, IN | 94 | 103 | 36 months | HSS | 1 | 1 | 1 | Improved from baseline, significance unclear; no significant difference between groups; Cumulative survival overall at 3 years is 97.1% or 90.7% when pain and aseptic loosening is considered | U |
| O’Rourke | 2002 | USA | TKA | SC | Cemented modular tibial component | Insall-Burstein, Zimmer | 62 | 87 | mean 6.4 | AKSS, HSS | 1 | 1 | 1 | Improved from baseline, significance unclear | F |
| Otte | 1997 | Denmark | TKA | SC | Results of revision of unicompartmental prostheses using cementless AGC | AGC prosthesis, Biomet, Warsaw, IN | 28 | 29 | median 38 months | HSS | 1 | 1 | 1 | Cementless revision comparable to cemented | U |
| Pagnano | 2004 | USA | TKA | RCT | Range of motion for polyethylene vs metal backed vs rotating platform tibia | Sigma Press Fit Condylar, DePuy | 240 | 240 | 1 | AKSS | 1 | 0 | 0 | No significant difference | F |
| Pandit | 2011 | UK | UKA | SC | Medial, minimally invasive | Oxford, Biomet | 818 | 1,000 | mean 5.6 | AKSS, OKS, TAS | 1 | 1 | 1 | Improved from baseline, 29 revisions | F |
| Pandit | 2006 | UK | UKA | SC | To determine complication rate, clinical outcome and midterm survival of minimally invasive approach | Oxford knee, Biomet, Bridgend, UK | 595 for survival; NR for clinical FU | 688 for survival; 101 for clinical FU | minimum 5 years for clinical FU | AKSS, OKS | 1 | 1 | 1 | Improved from baseline, significance unclear; Survival at 7 years is 97.3% | F |
| Pandit | 2013 | UK | UKA | RCT | Medial, Cement vs cementless | Oxford, Biomet | 62 | 63 | 5 (for 58 patients) | AKSS, OKS, TL | 1 | 1 | 0 | Significant improvement in only AKS for cementless | F |
| Pereira | 1998 | USA | TKA | CC | Posterior cruciate sparing vs retaining | Kinemax, Howmedica | 107 | 143 | mean 3 | HSS | 1 | 0 | 0 | No significant postoperative differences between groups | U |
| Pitson | 1994 | UK | TKA | SC | Change in quality of life | Minns Meniscal Knee | 26 | NR | 0.17, (at least 2 months) | Harris, McGill, NHP | 1 | 0 | 0 | Significantly improved from baseline | U |
| Price | 2005B | Sweden & UK | UKA | CC | To determine 10 year survival and clinical outcome of Oxford UKA in patients with anteromedia osteoarthritis <60 vs. > 60 | Oxford UKA, Biomet Ltd, Bridgend, UK | 447 | 564; 512 60 years or older, 52 younger than 60 years | minimum 10 years | HSS | 1 | 1 | 0 | HSS score better in younger group vs. older group, significance unclear; Survival at 10 years for younger group is 96% and for older group is 91% | F |
| Price | 2005A | Sweden | UKA | SC | Document the 15 year survival and 10 year clinical and radiological results of device | Oxford knee phase I, phase II and phase II, Biomet Ltd, Bridgend, UK | 351 for survival; 89 for clinical FU | 439 for survival; 114 for clinical FU | mean 10.5 years for clinical FU | HSS | 1 | 1 | 1 | Improved from baseline, significance unclear; Survival at 15 years is 93% | F |
| Pynsent | 2005 | UK | TKA | SC | Modified layout and scoring of OKS | NR | 1,458 | 1,739 | 4.2 | OKS | 1 | 0 | 0 | Improved at one year then did not change | I |
| Rajasekhar | 2004 | UK | UKA | SC | Outcome of Oxford UKA | Oxford knee phase II prosthesis, Biomet UK Ltd, Bridgend, UK | 124 | 135 | mean 5.82 | AKSS | 1 | 1 | 1 | No preop scores taken for AKS; Survival at 10 years is 94.04% | I |
| Rand | 1996 | USA | TKA | CC | Inset vs. resurfacing patellar prostheses | Genesis Prosthesis (Smith and Nephew Richard, Memphis, TEN) | 182 | 251 | Mean 2.3±0.5, (range 2-4) | AKSS, HSS | 1 | 1 | 1 | Significantly improved from baseline; Knee Society pain score was significantly higher for resurfacing group | U |
| Rand | 1991 | USA | TKA | CC | Cemented vs. cementless TKA | Press Fit Condylar, Johnson & Johnson Orthopaedics, Braintree, MA | 52 cemented; 50 cementless | 59 cemented; 59 cementless | mean 2.8, minimum 2 | AKSS, HSS | 1 | 1 | 1 | Both groups significantly improved from baseline; some significant differences between groups, but overall outcome is equivalent in both groups | U |
| Rasquinha | 2006 | USA | TKA | SC | Determine long-term results of PFC TKA after 12 years | Press fit condylar, Johnson and Johnson, Raynham, MA | 84 | 105 | mean 12 | AKSS, VAS | 1 | 1 | 1 | No preop scores taken for AKS; Survival at 12 years is 98.3% | I |
| Reay | 2009 | UK | TKA | CC | Comparision of Kinemax Plus (with high failure) vs. PFC Sigma | Kinemax Plus TKA, Stryker Howmedica Osteonics, Allendale, NJ; Press fit condylar TKA, Johnson & Johnson Professional Inc, Raynham, MA | 57 for Kinemax; 49 for PFC | NR for both | 76 months for Kinemax; 72 months for PFC | NR | 0 | 1 | 0 | No statistical difference between two groups identified as possible contributor to high rate of failure; Authors suspect manufacturing defect resulted in high failure rate of Kinemax | I |
| Rees | 2004 | UK | UKA | SC | Medial, minimally invasive | Oxford, Biomet | 91 | 104 | mean 1.3 | AKSS | 1 | 1 | 1 | Improved from baseline, significantly more so after learning curve of 10 cases | U |
| Regner | 1997 | Sweden | TKA | SC | Cementless tibial components fixed with finned polyethylene pegs | Freeman-Samuelson, Protek AG | 120 | 144 | mean 6.8, (for 88 patients) | HSS | 1 | 1 | 0 | Survival was 79% at 10 years, 17 revisions, this method of fixation not recommended | U |
| Rinta-Kiikka | 1999 | Finland | TKA | CC | Comparision between 2 contemporary cementless prostheses | Synatomic, Depuy, Warsaw, IN; AGC, Biomet, Warsaw, IN | 71 for Synatomic; 68 for AGC | 75 for Synatomic; 79 for AGC | mean 63 months for Synatomic; mean 50 for AGC | AKSS | 1 | 1 | 1 | No significant difference between two groups; AKS scores not declared | U |
| Rinta-Kiikka | 1996 | Finland | TKA | SC | Long-term follow-up (cementless) | Short-stemmed Synatomic VF Prosthesis (DePuy, Warzawa, Indiana) | 75 (8 died by 5-yr follow-up) | 102 | mean 5.3 (range 5-7) | AKSS | 1 | 1 | 1 | Significantly improved from baseline | U |
| Ritter | 1995b | USA | TKA | SC | Pre-op. vs post-op. function | NR | 158 | 223 | 2 | SF-36 | 1 | 0 | 1 | Improved significantly from baseline | U |
| Ritter | 2008 | USA | TKA | CC | Clinical effect of gender on outcome of TKA | AGC, Biomet, Warsaw, IN | NR; 59.5% female | 7326 | minimum 2 months, maximum 17 years | AKSS | 1 | 0 | 0 | Improvement after TKA is similar for both genders with few clinically significant differences | I |
| Ritter | 2003 | USA | TKA | CC | Unilateral vs bilateral vs staged bilateral | Anatomic Graduated Component, Biomet | 3,998 | 6,200 | mean 4.3 | AKSS | 1 | 1 | 1 | Bilateral had significantly higher scores than unilateral but higher rate of thrombo-phlebitis | I |
| Ritter | 2001 | USA | TKA | SC | To evaluate 15 year experience with AGC TKA | Anatomic graduated components, Biomet Inc, Warsaw, IN | 3054 | 4583 | mean 10.7 | AKSS | 1 | 1 | 0 | Survivorship at 15 years was 98.86%; AKS scores not declared and preop scores not taken | U |
| Rodricks | 2007 | USA | TKA | SC | 14 to 17 year results of PFC TKA | Press-fit condylar total knee prosthesis, Johnson and Johnson, Raynham, MA | 52 | 64 | mean 15.8 | AKSS | 1 | 1 | 1 | Improved from baseline, significance unclear; Survival overall was 91.5% at mean FU | I |
| Rodriguez | 2001 | USA | TKA | CC | To compare results of all-polyethylene tibia and the modular metal-backed tibia of the same cruciate-substituting design | Press-fit condylar; NR | 91 for all-polyethylene; 84 for metal-backed | 130 for all-polyethylene; 113 for metal-backed | 5.5 | AKSS | 1 | 1 | 1 | Significantly improved from baseline; No signficant difference between two methods of fixation | U |
| Rodriguez | 1996 | USA | TKA | SC | Long term follow-up | Total Condylar, Howmedica | 67 | 104 | mean 12.7 | AKSS, HSS | 1 | 1 | 1 | Improved from baseline, significance unclear | U |
| Rosenberg | 2001 | Australia | TKA | SC | Medium term results of cementless LCS in patients with osteoarthritis | cementless LCS mobile meniscal bearing posterior cruciate TKA, Depuy Orthopaedics Inc | 19 | 26 | 5 to 8 years | HSS, OKS | 1 | 1 | 1 | No preop HSS or OKS scores taken; Survival at 5 years is 97.1% | U |
| Sanchez-Sotelo | 1999 | Spain | TKA | SC | Midterm results of LCS knee prosthesis | LCS TKA, NR | 94 | 101 | mean 5.2 | AKSS | 1 | 1 | 1 | Improved from baseline, significance unclear | I |
| Sansone | 2004 | Italy | TKA | SC | Mobile total articulating cementless | TACK, Waldemar Link | 102 | 110 | 6.3 | AKSS | 1 | 1 | 1 | Improved from baseline, unclear significance | I |
| Santini | 2008 | UK | TKA | SC | Assessment of 10 year survival of first 99 TKAs performed by a surgeon | Press fit condylar TKA, Depuy International Ltd., Leeds, UK | 97 | 99 | mean 8 years and 8 months | NR | 0 | 1 | 1 | Survival at 10 years was 94.96% | I |
| Schail | 1998 | USA | TKA | SC | 10 year FU of PFC TKA | PFC Total Knee replacement system, Johnson & Johnson Professional Inc, Raynham, MA | 122 | 155 | mean 10.5 | AKSS | 1 | 1 | 1 | No preop AKS scores taken; Survivorship at 10 years is 90% | F |
| Schelfaut | 2012 | Belgium | UKA | SC | Lateral, Mobile | Oxford Domed Lateral, Biomet | 25 | 25 | 1 | OKS | 1 | 1 | 1 | Significantly improved from baseline | I |
| Schroder | 2001 | Denmark | TKA | SC | Cementless, porous-coated | Anatomically Graduated Component, Biomet | 52 | 58 | mean 10 | HSS | 1 | 1 | 1 | Findings unclear as reported | U |
| Schroder | 1996 | Denmark | TKA | SC | Medium term results and survivorship of primary cementless AGC | Anatomically graduated components, Biomet, Warsaw, IN | 32 | 41 | median 54 months | HSS | 1 | 1 | 1 | Preop HSS scores not taken; Survival at 4-5 years was 97% | U |
| Sextro | 2001 | USA | TKA | SC | Long term follow-up | Kinematic, Howmedica | 50 | 66 | mean 15.7 | AKSS | 1 | 1 | 1 | Improved from baseline, significance unclear | U |
| Sharma | 2005 | UK | TKA | SC | Long-term results of LCS TKA in patients with rheumatoid arthritis | Low contact stress mobile bearing knee replacement, Depuy Orthopaedics Inc., Warsaw, IN | 47 | 63 | mean 12.9 | AKSS | 1 | 1 | 1 | Preop AKS scores not taken; Survival at 16 years was 94% | I |
| Sharma | 1996 | USA | TKA | SC | Factors influencing outcome | NR | 47 | 47 | 0.25 | SF-36 | 1 | 0 | 1 | Significantly improved from baseline; social functioning associated with outcome | I |
| Shih | 2004 | Taiwan | TKA | SC | Patellar outcomes | Porous Coated Knee, Howmedica | 181 | 227 | mean 8.5 | AKSS | 1 | 0 | 0 | Improved from baseline, but significantly less so for those with preoperative patellar abnormalities | I |
| Song | 2009 | Korea | UKA | SC | Medial, minimally invasive | Oxford, Biomet | 94 | 100 | 2 | AKSS, WOMAC | 1 | 1 | 1 | Improved from baseline, 7 revisions | I |
| Sorrells | 2004 | USA | TKA | SC | Mobile rotating platform cementless | Low Contact Stress, DePuy | 282 | 371 | mean 8.25 | NJOH | 1 | 1 | 1 | Improved at one year then did not change | F |
| Sorrells | 2001 | USA | TKA | SC | Results of mobile-bearing TKA offered better outcome for patients younger than 65 | LCS rotating platform total knee prosthesis, Depuy/Johnson & Johnson, Warsaw, IN | 74 | 91 | mean 8.5 | NJOH | 1 | 1 | 1 | Improved from baseline, significance unclear; Survival at 14 years is 94.6% | U |
| Sorrells | 1996 | USA | TKA | SC | Clinical results of non-cemented TKA after 11 years | New Jersey LCS TKA, Depuy, Warsaw, IN | 521 | 665 | 1 to 11 years | NJOH | 1 | 1 | 1 | HSS scores not declared; Survival at 11 years is 94.7% | U |
| Starks | 2009 | UK | UKA | SC | Patellofemoral, Assess outcomes | Avon, Stryker | 29 | 37 | mean 2 | AKSS, Melbourne, OKS | 1 | 0 | 1 | No comparator | I |
| Stickles | 2001 | USA | TKA | CC | Outcomes among healthy vs overweight vs class I, II and III obese patients | NR | 1,011 | NR | 1 | SF-36, WOMAC | 1 | 0 | 1 | Improved from baseline, no significant difference between groups | F |
| Stockley | 1988 | UK | TKA | SC | Experience with St. George sledge used in bicondylar surface replacement operations | St. Georg sledge prosthesis; NR | 35 | 44 | mean 5.9 | D'Aubigné | 1 | 1 | 1 | Improved from baseline, significance unclear | U |
| Stukenborg-Colsman | 2001 | Germany | UKA Only | RCT | UKA (medial) vs HTO | UKA Unicondylar knee sliding prosthesis, Tubingen pattern, Aesculap-Orthec | 28 | 30 | mean 7.5 | AKSS | 1 | 1 | 1 | UKA - Improved from baseline | U |
| Sun | 2012 | China | TKA, UKA | RCT | Medial, UKA vs TKA | Oxford, Biomet | 56 | 56 | mean 4.3 | AKSS | 1 | 1 | 1 | No significant difference between groups, 7 UKA revisions | I |
| Svard | 2001 | Sweden | UKA | SC | Long term survival rate of Oxford Knee in patients with anteromedial osteoarthritis | Oxford Knee Phase I and Phase II, Biomet Ltd., Bridgend, UK | 60 | 74 | mean 12.5 | NR | 0 | 1 | 1 | Survival at 16 years is 95% | F |
| Tarkin | 2005 | USA | TKA | SC | To determine the long term results of rotating-platform total knee implanted cementlessly | Porocoat low contact stress, Depuy, Warsaw, IN | NR | 23 | mean 15.5 | HSS | 1 | 1 | 1 | Improved from baseline, significance unclear; Survival at 17 years was 97% | I |
| Therbo | 2003 | Denmark | TKA | SC | Assess whether preoperative bone mineral density associated with revision rate | Porous-coated anatomic TKA, Howmedica; Press-fit condylar TKA, Johnson & Johnson Orthopaedics | 106 | 106 | NR | NR | 0 | 1 | 1 | Preoperative bone mineral density not associated with revision rate | I |
| Tibesku | 2011 | Germany | TKA | RCT | Fixed vs. mobile bearing | Genesis II, either fixed or mobile-bearing (Smith and Nephew) | 33 | NR | Mean 2 (fixed group); Mean 23.8 months (mobile group) | AKSS, HSS | 1 | 0 | 0 | Significantly improved from baseline; KSS score significantly better for mobile group | U |
| Title | 2001 | USA | TKA | CC | Cruciate sacrificing vs cruciate substituting, same surgical approach | Total Condylar, Howmedica; Press Fit Condylar, Johnson & Johnson | 148 | 168 | mean 4.2 | AKSS | 1 | 1 | 0 | Improved from baseline, PFC outcomes were significantly greater | I |
| Verdonk | 2005 | Belgium | UKA | SC | 2-14 year follow up of Oxford UKA | Oxford unicompartmental knee prosthesis, Biomet Merck, Dordrecht, Netherlands | 69 | 69 | mean 81 months | HSS | 1 | 1 | 1 | Preop HSS scores not taken, improvement unclear | U |
| Ververeli | 1995 | USA | TKA | CC | Added benefit of continuous passive motion on physical therapy | Anatomic Graduated Component, Biomet | 103 | NR | 2, (for 97 patients) | HSS | 1 | 1 | 1 | Significantly improved from baseline; no significant difference between groups | U |
| Vessely | 2006 | USA | TKA | SC | Survivorship of cemented condylar cruciate-retaining TKAs | cemented cruciate-retaining press-fit condylar TKA, Depuy/Johnson and Johnson, Warsaw, IN | 244 | 331 | 15.7 | NR | 0 | 1 | 1 | Survivorship overall at 15 years is 93.7% | U |
| Vorlat | 2006 | Belgium | UKA | SC | Long-term followup of Oxford UKA at three hospitals | Oxford unicompartmental knee prostheses, Biomet Merck, Swindon, UK | 17 | 18 | mean 126 months | AKSS at 2/3 hospitals, only thorough history taken at one hospital | 1 | 1 | 1 | No preop AKS scores taken; Survivorship at 10 years is 82% | U |
| Vorlat | 2000 | Belgium | UKA | SC | Followup after 5 years for Oxford UKA | Oxford unicompartmental knee prostheses, Depuy, Leeds | 39 | 41 | mean 58 months | HSS | 1 | 1 | 1 | No preop HSS scores taken, results unclear | U |
| Watanabe | 2005 | Japan | TKA | RCT | Fixed vs. mobile bearing | Rotaglide for mobile-bearing (Corin, UK) or NexGen CR for fixed-bearing (Zimmer, USA) | 22 | 44 | Mobile group mean 98 months, fixed group mean 96 months | AKSS | 1 | 0 | 1 | Improved from baseline, unclear significance; No significant difference between groups | U |
| Waters | 2003 | UK | TKA | RCT | Patellar resurfacing vs retention | Press Fit Condylar, Johnson & Johnson | 390 | 474 | mean 5.3 | AKSS | 1 | 0 | 0 | No significant difference | F |
| Weale | 1994 | UK | UKA Only | CC | UKA (medial) vs HTO | UKA St. Georg Sled, Waldemar Link | 34 | 42 | 6, (for 36 patients) | Baily | 1 | 1 | 0 | UKA - Improved from baseline | U |
| Weaver | 1993 | USA | TKA | CC | Cemented vs. cementless in LCS TKA | New Jersey/LCS, Depuy, Warsaw, IN | 40 cemented; 16 cementless | 40 cemented; 16 cementless | mean 85 months for cemented, mean 59 months for cementless | NJOH | 1 | 1 | 1 | Both groups improved from baseline, significance unclear; cemented group had failure rate of 10%, cementless group had failure rate of 31% | U |
| Weber | 2002 | USA | TKA | CC | Monoblock vs. modular tibial component in AGC TKA | AGC, Biomet , Warsaw, IN | 757 | 1051 total; 698 monoblock, 353 modular | 5 to 11 years | HSS | 1 | 1 | 1 | Both groups improved from baseline, significance unclear; No significant clinical differences between groups, though monoblock gave less revisions | I |
| Westwood | 2003 | UK | TKA | CC | To determine whether there is a significant difference between St. Leger and Kinemax Plus TKRs | St. Ledger TKR, NR; Kinemax Plus TKR, NR | 34 for St. Ledger; 36 for Kinemax | 38 for St. Ledger; 38 for Kinemax | mean 70.32 months for St. Ledger; mean 81.57 months for Kinemax | OKS | 1 | 1 | 1 | No significant clinical difference between two groups, though St. Ledger had more revisions; Survival rate of Kinemax is 100% and St. Ledger is significantly worse at 91.2% | U |
| Woolson | 2004 | USA | TKA | RCT | Mobile vs. Fixed bearing in TKA performed by one surgeon | Mobile-bearing LCS rotating platform, Depuy, Warsaw, IN; Fixed-bearing Nex Gen PS, Zimmer, Warsaw, IN | 44 for mobile; 40 for fixed | 57 for mobile; 45 for fixed | mean 41 months for mobile; mean 43 months for fixed | AKSS | 1 | 1 | 1 | Preop scores for AKS not declared; No significant differences between groups though fixed has less revisions | I |
| Worland | 2002 | USA | TKA | SC | To evaluate the survival of single posterior cruciate-retaining system | Anatomic graduated component, NR | 145 | 212 | minimum 10 years, range 10-14 years | AKSS | 1 | 1 | 1 | AKS scores not declared; Survival at 14 years was 97% | U |
| Worland | 1998 | USA | TKA | RCT | At-home physical therapy vs continuous passive motion upon discharge | Anatomic Graduated Component, Biomet or Foundation Knee, Encore | 80 | 103 | 0.5 | HSS | 1 | 0 | 0 | Significantly improved from baseline; no significant difference between groups | U |
| Wright | 2004 | USA | TKA | SC | Survival rate of Kinemax TKA after 10 years | Kinemax Prosthesis, Stryker Howmedica Osteonics, Allendale, NJ | 198 | NR | mean 11.7 | KOOS, Patient Satisfaction, SF-36, WOMAC | 1 | 1 | 1 | Improved from baseline (KOOS), significance unclear; No preop scores taken for SF-36 and WOMAC | F |
| Wright | 1990 | USA | TKA | SC | To determine if a hybrid cementless TKA could equal results of cemented TKA (in other literature) | Press-fit condylar posterior cruciate-sparing knee system, Johnson & Johnson, Braintree, MA | 99 | 114 | mean 2.8 | AKSS, Brigham | 1 | 1 | 1 | Improved from baseline, significance unclear; Hybrid TKA provides comparable results to cemented TKA | U |
| Yang | 2003 | Singapore | TKA, UKA | CC | UKA (medial) vs TKA | UKA PFC, DePuy or Miller-Galante, Zimmer; TKA NexGen, Zimmer or PFC Sigma, DePuy | 100 | 100 | 0.5 | NR | 0 | 0 | 1 | Days to ambulation, LOS and range of motion improved significantly in UKA group | U |
| Yang | 2001 | Singapore | TKA | SC | Outcomes among diabetic patients | NR | 86 | 109 | mean 3.5 | AKSS | 1 | 1 | 1 | Significantly improved from baseline | I |
| Zaki | 2007 | UK | TKA | SC | Mid-term results of PFC Sigma knee arthroplasties | Press-fit condylar total knee prosthesis, Johnson and Johnson, Raynham, MA | 145 | 155 | mean 90 months | AKSS, OKS | 1 | 1 | 1 | Improved from baseline, significance unclear | I |

REFERENCES

(1) Ackroyd CE, Whitehouse SL, Newman JH, Joslin CC. A comparative study of the medial St George sled and kinematic total knee arthroplasties. Ten-year survivorship. J Bone Joint Surg Br 2002;84(5):667-672.

(2) Adalberth G, Nilsson KG, Bystrom S, Kolstad K, Milbrink J. Low-Conforming All-Polyethylene Tibial Component Not Inferior to Metal-Backed Compoment in Cemented Total Knee Arthroplasty. J Arthroplasty 2000;15(6):783-792.

(3) Aebli N, Krebs J, Schwenke D, Hii T, Wehrli U. Progression of radiolucent lines in cementless twin-bearing low-contact-stress knee prostheses. J Arthroplasty 2004;19(6):783-789.

(4) Aigner C, Windhager R, Pechmann M, Rehak P, Engeleke K. The influence of an anterior-posterior gliding mobile bearing on range of motion after total knee arthroplasty. J Bone Joint Surg Am 2004;86(10):2257-2262.

(5) Alemparte J, Cabezas A, Azocar O, Hernandez R, Acevedo M. Mid-term results of an AGC total knee arthroplasty system survival and function analysis. J Arthroplasty 2003;18(4):420-425.

(6) Ali MS, Mangaleshkart SR. Uncemented rotating-platform total knee arthroplasty. A 4-year to 12-year follow-up. J Arthroplasty 2006;21(1):80-84.

(7) Ansari S, Newman JH, Ackroyd CE. St.Georg sledge for medical compartment knee replacement. Acta orthrop scand 1997;68(5):430-434.

(8) Ashraf T, Newman JH, Evans RL, Ackroyd CE. Lateral unicompartmental knee replacement. J Bone Joint Surg Am 2002;84(B):1126-30.

(9) Asif S, Choon DSK. Midterm results of cemented press fit condylar sigma total knee arthroplasty system. J Orthop Surg 2005;13(3):280-284.

(10) Attar FG, Khaw FM, Kirk LMG, Gregg PJ. Survivorship analysis at 15 years of cemented press-fit condylar total knee arthroplasty. J Arthroplasty 2008;23(3):344-349.

(11) Bachmeier C, March L, Cross M. A comparison of outcomes in osteoarthrirtis patients undergoing total hip and knee replacment surgery. Osteoarthritis Cartilage 2001;9(2):137-146.

(12) Back DL, Cannon SR, Hilton A, Bankes MJK, Briggs TWR. The kinemax total knee arthroplasty. J Bone Joint Surg 2001;83-B(3):359-363.

(13) Bailey O, Fergusson K, Crawford E, James P, May PA, Brown S. No clinical difference between fixed-and mobile-bearing cruciate retaining total knee arthroplasty: a prospective randomized study. Knee Surg Sports Traumatol Arthrosc 2014:Epub.

(14) Baker PN, Khaw FM, Kirk LMG, Esler CNA, Gregg PJ. A randomised controlled trial of a cemented versus cementless press-fit condylar total knee replacement. J Bone Joint Surg Am 2007;89-B(12):1608-1614.

(15) Baldwin J, Rubinstein RJ. The effect of bone quality on the outcome of ingrowth total knee arthroplasty. Am J Knee Surg 1996;9(2):45-49.

(16) Ballantyne A, McKinley J, Brenkel I. Comparison of the lateral release rates in the press fit condylar prosthesis and the PFC sigma prosthesis. The Knee 10 2003:193-198.

(17) Bankes MJ, Back DL, Cannon SR, Briggs TW. The effect of component malalignment on the clinical and radiological outcome of the Kinemax total knee replacement. Knee 2003;10(1):55-60.

(18) Beard DJ, Pandit H, Gill HS, Hollinghurst D, Dodd CA, Murray DW. The influence of the presence and severity of pre-existing patellofemoral degenerative changes on the outcome of the Oxford medial unicompartmental knee replacement. J Bone Joint Surg Br 2007;89(12):1597-1601.

(19) Beard DJ, Pandit H, Price AJ, Butler-Manuel PA, Dodd CAF, Murray DW, et al. Introduction of a new mobile-bearing total knee prosthesis - Minimum three year follow-up of an RCT comparing it with a fixed-bearing device. The Knee 14 2007:448-451.

(20) Beaupre L, Davies D, Jones C. Exercise combined with continuous passive motion or slider board therapy compared with exercise only: a randomized controlled trial of patients following total knee arthroplasty. Phys Ther 2001;81(4):1029-1037.

(21) Beaupre LA, Al-Yamani M, Huckell JR, Johnston DWC. Hydroxyapatite-coated tibial implants comparted with cemented tibial fixation in primary total knee arthroplasty. A randomized trial of outcomes at five years. J Bone Joint Surg 2007;89(10):2204-2211.

(22) Berend KR, Lombardi AVJ, Adams JB. Obesity, young age, patellofemoral disease, and anterior knee pain: identifying the unicondylar arthroplasty patient in the United States. Orthopedics 2007;30(5-Supplement):19-23.

(23) Berend ME, Ritter MA, Hyldahl HC, Meding JB, Redelman R. Implant migration and failure in total knee arthroplasty is related to body mass index and tibial component size. J Arthroplasty 2008;23(6 Suppl. 1):104-109.

(24) Bert J, Gross M, Kline C. Patient demand matching in total knee arthroplasty: is it necessary? Am J Knee Surg 2001;14(1):39-42.

(25) Bert J, Gross M, Kline C. Outcome results after total knee arthroplasty: does the patient's physcial and mental health improve? Am J Knee Surg 2000;13(4):223-227.

(26) Bertin KC. Cruciate-retaining total knee arthroplasty at 5 to 7 years followup. Clin Orthop Relat Res 2005;436:177-183.

(27) Bhan S, Malhotra R. Results of rotating-platform, Low-contact-stress knee prosthesis. J Arthroplasty 2003;18(8):1016-1022.

(28) Bhan S, Malhotra R, Kiran EK, Shukla S, Bijjawara M. A comparison of fixed-bearing and mobile-bearing total knee arthroplasty at a minimum follow-up of 4.5 years. J Bone Joint Surg Am 2005;87(10):2290-2296.

(29) Bietzel K, Schottle PB, Cotic M, Dharmesh V, Imhoff AB. Prospective clinical and radiological two-year results after patellofemoral arthroplasty using an implant with an asymmetric trochlea design. Knee Surg Sports Traumatol Arthrosc 2013;21:332-339.

(30) Birdsall PD, Hayes JH, Cleary R, Pinder IM, Moran CG, Sher JL. Health outcome after total knee replacement in the very elderly. J Bone Joint Surg Br 1999;81:660-662.

(31) Biswas D, VanThiel GS, Wetters NG, Pack BJ, Berger RA, DeliaValle CJ. Medical unicompartmental knee arthroplasty in patients less than 55 years old: minimum of two years of follow-up. J Arthroplasty 2013.

(32) Borjesson M, Weidenhielm L, Mattsson E, Olsson E. Gait and clinical measurements in patients with knee osteoarthritis after surgery; a prospective 5-year follow-up study. The Knee - in press 2004.

(33) Boublik M, Tsahakis PJ, Scott RD. Cementless total knee arthroplasty in juvenile onset rheumatoid arthritis. Clin Orthop Relat Res 1993;286:88-93.

(34) Bozic KJ, Kinder J, Manegini M, Zurakowski D, Rosenberg AG, Galante JO. Implant survivorship and complication rates after total knee arthroplasty with a third-generation cemented system. Clin Orthop Relat Res 2005;430:117-124.

(35) Brander VA, Stulberg SD, Adam AD, Harden RN, Bruhel S, Stanos SP. Predicting total knee replacement pain: a prospective, observational study. Clin Orthop 2003;416:27-36.

(36) Brazier JE, Harper R, Munro J, Walters SJ, Snaith ML. Generic and condition-specific outcome measures for people with osteoarthritis of the knee. Rheumatology (Oxford) 1999;38:870-877.

(37) Brown T, Diduch D, Moskal J. Component size asymmetry in bilteral total knee arthroplasty. Am J Knee Surg 2001;14(2):81-84.

(38) Bruni D, Akkawi I, Iacono F, Raspugli GF, Gagliardi M, Nitri M. Minimum thickness of all-poly tibial component unicompartmental knee arthroplasty in patients younger than 60 years does not increase revision rate for aseptic loosening. Knee Surg Sports Traumatol Arthrosc 2013.

(39) Buechel FF, Pappas MJ. The new jersey low-contact-stress knee replacement system: biomechanical rationale and review of the first 123 cemented cases. Arch Orthop Trauma Surg 1986;105:197-204.

(40) Buechel FFS. Long-term followup after mobile-bearing total knee replacement. Clin Orthop Relat Res 2002;404:40-50.

(41) Buechel FFS, Buechel FF, Pappas MJ, D'Alessio J. Twenty-Year evaluation of meniscal bearing and rotating platform knee replacements. Clin Orthop Relat Res 2001;388:41-50.

(42) Bullens P, VanLoon C, deWaal-Malefijt M. Patient satisfaction after total knee arthroplasty: a comparison between subjective and objective outcome assessments. J Arthroplasty 2001;16(6):740-747.

(43) Callaghan JJ, O'Rourke MR, Iossi MF, Liu SS, Goetz DD, Vittetoe DA, et al. Cemented rotating-plaftorm total knee replacment: A concise follow-up, at a minimum of fifteen years, of a previous report. J Bone Joint Surg Am 2005;87-A(9):1995-1998.

(44) Cameron HU, Jung YB. A comparison of unicompartmental knee replacement with total knee replacement. Orthopaedic Review 1988;17(10):983-988.

(45) Carlsson A, Bjorkman A, Besjakov J, Onsten I. Cemented tibial component fixation performs better than cementless fixation.  A randomized radiostereometric study comparing porous-coated, hydroxyapatite-coated and cemented tibial components over 5 years. Acta Orthop 2005;76(3):362-369.

(46) Carr A, Keys G, Miller R, O'Connor J, Goodfellow J. Medial unicompartmental arthroplasty. A Survival Study of the Oxford Meniscal Knee. Clin Orthop Relat Res 1993;295:205-213.

(47) Chiu KY, Ng TP, Tang WM, Lam P. Bilateral total knee arthroplasty: One mobile-bearing and one fixed-bearing. J Orthop Surg 2001;9(1):45-50.

(48) Choy WS, Kim KJ, Lee SK, Yang DS, Lee NK. Mid-term results of oxford medial unicompartmental knee arthroplasty. Clin Orthop Surg 2011;3(3):178-183.

(49) Christensen NO. Unicompartmental prosthesis for gonarthrosis.  A nine-year series of 575 knees from a Swedish Hospital. Clin Orthop Relat Res 1991;273:165-169.

(50) Clark C, Rorabeck C, MacDonald S. Posterior-stabilized and cruciate-retaining total knee replacement: a randomized study. Clin Orthop 2001;392:208-212.

(51) Clayton RAE, Amin AK, Gaston MS, Brenkel IJ. Five-year results of the Sigma total knee arthroplasty. The Knee 13 2006:359-364.

(52) Clement ND, Duckworth AD, MacKenzie SP, Nie YX, Tiemessen CH. Medium-term results of Oxford phase-3 medial unicompartmental knee arthroplasty. J Orthop Surg 2012;20(2):157-161.

(53) Cloke DJ, Khatri M, Pinder IM, McCaskie AW, Lingard EA. 284 press-fit Kinemax total knee arthroplasties followed for 10 years. Acta Orthop 2008;79(1):28-33.

(54) Cloutier J, Sabouret P, Deghrar A. Total knee arthroplasty with retention of both cruciate ligaments. A 9 to 11 year follow-up study. Eur J Orthop Surg Traumatol 2001;11(1):41-46.

(55) Cohen R, Forrest C, Benjamin J. Safety and efficacy of bilateral total knee arthroplasty. J Arthroplasty 1997;12(5):497-502.

(56) Confalonieri N, Manzotti A, Pullen C. Comparison of a mobile with a fixed tibial bearing unicompartmental knee prosthesis; a prospective randomized trial using a dedicated outcome score. Knee 2004;11:357-362.

(57) Cool S, Victor J, DeBaets T. Does a minimally invasive approach affect positioning of components in unicompartmental knee arthroplasty? Early results with survivorship analysis. Acta Orthop Belg 2006;72(6):709-715.

(58) Dalury DF, Barrett WP, Mason JB, Goldstein WM, Murphy JA, Roche MW. Miderm survival of a contemporary modular total knee replacement. J Bone Joint Surg Am 2008;90-B(12):1594-1596.

(59) Davis CR, Davies AP, Newman JH. Causes of failure of the kinemax plus total knee replacment in the first five years. The Knee 2007;14:158-161.

(60) Dawson J, Fitzpatrick R, Murray D, Carr A. Questionnaire on the perceptions of patients about total knee replacement. J Bone Joint Surg Br 1998;80:63-69.

(61) Deshmukh R, Hayes J, Pinder I. Does body weight influence outcome after total knee arthroplasty? A 1-year analysis. J Arthroplasty 2002;17(3):315-319.

(62) Diduch D, Insall J, Scott W. Total knee replacement in young, active patients. Long-term follow-up and functional outcome. J Bone Joint Surg Am 1997;79(4):575-582.

(63) Dixon MC, Brown R, Parsch D, Scott RD. Modular fixed-bearing total knee arthroplasty with retention of the posterior cruciate ligament. J Bone Joint Surg Am 2005;87(3):598-603.

(64) Donell ST, Neyret P, Dejour H, Adeleine P. The effect of age on the quality of life after knee replacement. Knee 1998;5:125-128.

(65) Duffy G, Berry D, Rand J. Cement versus cementless fixation in total knee arthroplasty. Clin Orthop 1998;356:66-72.

(66) Duffy GP, Crowder AR, Trousdale RR, Berry DJ. Cemented total knee arthroplasty using a modern prosthesis in young patients with osteoarthritis. J Arthroplasty 2007;22(6):67-70; Suppl. 2.

(67) Duffy GP, Murray BE, Trousdale RR. Hybrid total knee arthroplasty.  Analysis of component failures at an average of 15 years. J Arthroplasty 2007;22(8):1112-1115.

(68) Elke R, Meier G, Warnke K. Outcome analysis of total knee-replacements in patients with rheumatoid arthritis versus osteoarthritis. Arch Orthop Trauma Surg 1995;114(6):330-334.

(69) Emerson RHJ, Higgins LL. Unicompartmental knee arthroplasty with the Oxford prosthesis in patients with medial compartmental arthritis. J Bone Joint Surg Am 2008;90:118-122.

(70) Emerson RH, Hansborough T, Reitman RD, Rosenfeldt W, Higgins LL. Comparison of a mobile with a fixed-bearing unicompartmental knee iimplant. Clin Orthop Relat Res 2002;404:62-70.

(71) Emerson RHJ, Higgins LL. A comparison of highly instrumented and minimally instrumented unicompartmental knee prostheses. Clin Orthop Relat Res 2004;428:153-157.

(72) Emerson RHJ, Higgins LL, Head WC. The ACG total knee prosthesis at average 11 years. J Arthroplasty 2000;15(4):418-423.

(73) Evanich C, Tkach T, VonGlinski S. 6- to 10-year experience using countersunk metal-backed patellas. J Arthroplasty 1997;12(2):149-154.

(74) Ewald F, Wright R, Poss R. Kinematic total knee arthroplasty. A 10- to 14-year prospective follow-up review. J Arthroplasty 1999;14(4):473-480.

(75) Faris PM, Keating EM, Farris A, Meding JB, Ritter MA. Hybrid total knee arthroplasty. 13-year survivorship of AGC total knee systems with average 7 years followup. Clin Orthop Relat Res 2008;466:1204-1209.

(76) Faris PM, Ritter MA, Aleto TJ, Pierce AL. A comparison of the PCL-retaining AGC and posterior stabilizing legacy prostheses for total knee arthroplasty. HSSJ 2006;2:127-129.

(77) Faris PM, Ritter MA, Keating EM, Meding JB, Harty LD. The AGC all-polyethylene tibial component: A ten-year clinical evaluation. J Bone Joint Surg Am 2003;85(3):489-493.

(78) Fetzer GB, Callaghan JJ, Templeton JE, Goetz DD, Sullivan PM, Kelley SS. Posterior cruciate-retaining modular total knee arthroplasty. J Arthroplasty 2002;17(8):961-966.

(79) Fitzgerald JD, Orav EJ, Lee TH, Marcantonio ER, Poss R, Goldman L. Patient quality of life during the 12 months following joint replacement surgery. Arthritis Rheum 2004;51(1):100-109.

(80) Forster MC, Bauze AJ, Keene GCR. Lateral unicompartmental knee replacement; fixed or mobile bearing? Knee Surg Sports Traumatol Arthrosc 2007;15:1107-1111.

(81) Fortin P, Clarke A, Joseph L, et al. Outcomes of total hip and knee replacement: preoperative functional status predicts outcomes at six months after surgery. Arthritis Rheum 1999;42(8):1722-1728.

(82) Geiger F, Mau H, Kruger M, Thomsen M. Comparison of a new mobile-bearing total knee prosthesis with a fixed-bearing prosthesis: a matched pair analysis. Arch Orthop Trauma Surg 2008;128:285-291.

(83) Gill G, Joshi A. Long-term results of cemented, posterior cruciate ligament-retaining total knee arthroplasty in osteoarthritis. Am J Knee Surg 2001;14(4):209-214.

(84) Gill G, Joshi A, Mills D. Total condylar knee arthroplasty. 16- to 21-year results. Clin Orthop 1999;367:210-215.

(85) Gioe T, Bowman K. A randomized comparison of all-polyethylene and metal-backed tibial components. Clin Orthop 2000;380:108-115.

(86) Gleeson RE, Evans R, Ackroyd CE, Webb J, Newman JH. Fixed or mobile bearing unicompartmental knee replacement? A comparative cohort study. Knee 2004;11(5):379-384.

(87) Goldberg VM, Kraay M. The outcome of the cementless tibial component: a minimum 14-year clinical evaluation. Clin Orthop 2004;428(214-220).

(88) Goodfellow JW, Kershaw CJ, Benson MKD, O'Connor JJ. The oxford knee for unicompartmental osteoarthritis. J Bone Joint Surg Am 1988;70-B(5):692-701.

(89) Griffin F, Scuderi G, Insall J. Total knee arthroplasty in patients who were obese with 10 years followup. Clin Orthop 1998;356:28-33.

(90) Griffin WL, Scott RD, Dalury DF, Mahoney OM, Chiavetta JB, Odum SM. Modular insert exchadnge in knee arthroplasty for treatment of wear and osteolysis. Clin Orthop Relat Res 2007;464:132-137.

(91) Griffiths G, Bellamy N, Bailey WH, Bailey SI, McLaren AC, Campbell J. A comparative study of the relative efficiency of the WOMAC, AIMS, and HAQ instruments in evaluating the outcome of total knee arthroplasty. Inflammopharmacology 1995;3:1-6.

(92) Gupta SK, Ranawat AS, Shah V, Zikria BA, Zikria JF, Ranawat CS. The P.F.C. Sigma RP-F TKA Designed for improved performance: a matched-pair study. Orthopedics 2006;29(9-Supplement):S49-S52.

(93) Hansson U, Toksvig-Larsen S, Jorn LP, Ryd L. Mobile vs. fixed meniscal bearing in total knee replacement: a randomised radiostereometric study. The Knee 2005;12(6):414-418.

(94) Hanush B, Lou TN, Warriner G, Hui A, Gregg P. Functional outcome of PFC sigma fixed and rotating-platform total knee arthroplasty. A prospective randomised controlled trial. International Orthopaedics 2010;34:349-354.

(95) Hartford JM, Hunt T, Kaufer H. Low contact stress mobile bearing total knee arthroplasty. J Arthroplasty 2001;16(8):977-983.

(96) Harwin S. Patellofemoral complications in symmetrical total knee arthroplasty. J Arthroplasty 1998;13(7):753-762.

(97) Hasegawa M, Ohashi T, Uchida A. Heterotopic ossification around distal femur after total knee arthroplasty. Arch Orthop Trauma Surg 2002(122):5-274-278.

(98) Hassaballa MA, Porteous AJ, Newman JH, Rogers CA. Can knees kneel? Kneeling ability after total, unicompartmental and patellofemoral knee arthroplasty. Knee 2003;10(2):155-160.

(99) Healy W, Iorio R, Ko J. Impact of cost reduction programs on short-term patient outcome and hospital cost of total knee arthroplasty. J Bone Joint Surg Am 2002;84-A(3):348-353.

(100) Heck D, Robinson R, Partridge C. Patient outcomes after knee replacement. Clin Orthop 1998;356:93-110.

(101) Heller S, Fenichel I, Salai M, Luria T, Velks S. The oxford unicompartmental knee prosthesis for the treatment of medial compartment knee disease: 2 to 5 year follow-up. Isr Med Assoc J 2009;11(5):266-268.

(102) Henricson A, Dalen T, Nilsson KG. Mobile bearings do not improve fixation in cemented total knee arthroplasty. Clin Orthop Relat Res 2006;448:114-121.

(103) Himanen AK, Belt EA, Lehto MUK, Hamalainen MMJ. A comparison of survival of moulded monoblock and modular tibial components of 751 AGC total knee replacements in the treatment of rheumatoid arthritis. J Bone Joint Surg Am 2007;89-B(5):609-614.

(104) Hirsch HS, Lotke PA, Morrison LD. The posterior cruciate ligament in total knee surgery. Save, Sacrifice, or Substitute? Clin Orthop Relat Res 1994;309:64-68.

(105) Hooper GJ, Maxwell AR, Wilkinson B, Mathew J, Woodfeld TB, Penny ID. The early radiological results of the uncemented Oxford medial compartment knee replacement. J Bone Joint Surg Br 2012;94(3):334-338.

(106) Hsu R, Tsai Y, Huang T. Hybrid total knee arthroplasty: a 3- to 6-year outcome analysis. J Formos Med Assoc 1998;97(6):410-415.

(107) Huang CH, Liau JJ, Ho FY, Lin CY, Young TH, Cheng CK. Polyethylene failure of the patellar component in New Jersey low-contact stress total knee arthroplasties. J Arthroplasty 2005;20(2):202-208.

(108) Huang CH, Ma HM, Lee YM, Ho FY. Long-term results of low contact stress mobile-bearing total knee replacements. Clin Orthop Relat Res 2003;416:265-270.

(109) Hyldahl H, Regner L, Carlsson L, Karrholm J, Weidenhielm L. All-polyethylene vs. metal-backed tibial component in total knee arthroplasty-a randomized RSA study comparing early fixation of horizontally and completely cemented tibial components. Acta Orthop 2005;76(6):769-777.

(110) Hyldahl H, Regner L, Carlsson L, Karrholm J, Weidenhielm L. All-polyethylene vs. metal-backed tibial component in total knee arthroplasty-a randomized RSA study comparing early fixation of horizontally and completely cemented tibial components. Acta Orthop 2005;76(6):778-784.

(111) Ikejiani C, Leighton R, Pierre D. Comparison of patellar resurfacing versus nonresurfacing in total knee arthroplasty. Can J Surg 2000;43(1):35-38.

(112) Indelli P, Aglietti P, Buzzi P. The Insall-Burnstein II prosthesis: A 5- to 9-year follow-up study in osteoarthritic knees. J Arthroplasty 2002;17(5):544-549.

(113) Ivarson I, Gillquist J. Rehabilitation after high tibial osteotomy and unicompartmental arthroplasty. A comparative study. Clin Orthop Relat Res 1991;266:139-144.

(114) Jacobs WCH, Christen B, Wymenga AB, Schuster A, VanderSchaaf DB. Functional performance of mobile versus fixed bearing total knee prostheses: a randomised controlled trial. Knee Surg Sports Traumatol Arthrosc 2011;20:1450-1455.

(115) Jahromi I, Walton NP, Dobson PJ, Lewis PL, Campbell DG. Patient-perceived outcome measures following unicompartmental knee arthroplasty with mini-incision. Int Orthop 2004:1-4.

(116) Jain RK, Neville LT, Ezzet KA, Sterling RS, Horwood RL, Colwell CWJ. Two year follow-up of the preservation unicompartmental knee implant. HSS J 2011;7(2):125-129.

(117) Jeer PJS, Keene GCR, Gill P. Unicompartmental knee arthroplasty: an intermediate report of survivorship after the introduction of a new system with analysis of failures. The Knee 11 2004:369-374.

(118) Jenny J, Jenny G. Preservation of anterior cruciate ligmament in total knee arthroplasty. Arch Orthop Trauma Surg 1998;118(3):145-148.

(119) Jones CA, Voaklander DC, Johnston DW. The effect of age on  pain, function, and quality of life after total hip and knee arthroplasty. Arch Intern Med 2001;161(3):454-460.

(120) Jones CA, Voaklander DC, Johnston DW, Suarez-Almazor ME. Health related quality of life outcomes after total hip and knee arthroplasties in a community based population. J Rheumatol 2000;27:1745-1752.

(121) Jones CA, Voaklander DC, Suarez-Alma ME. Determinants of fuction after total knee arthroplasty. Phys Ther 2003;83(8):696-706.

(122) Jordan L, Olivo J, Voorhorst P. Survivorship analysis of cementless meniscal bearing total knee arthroplasty. Clin Orthop 1997;338:119-123.

(123) Joshi AB, Markovic L, Gill G. Knee arthroplasty in octogenarians: results at 10 years. J Arthroplasty 2003;18(3):295-298.

(124) Kageyama Y, Miyamoto S, Ozeki T, Hiyoshi M, Kushida K, Inoue T. Outcomes for patients undergoing one or more total hip and knee arthroplasties. Clin Rheumatol 1998;17:130-134.

(125) Kasodekar VB, Yeo SJ, Othman S. Clinical outcome of unicompartmental knee arthroplasty and influence of alignment on prosthesis survival rate. Singapore Med J 2006;47(9):796-802.

(126) Katz JN, Wright EA, Guadagnoli E, Liang MH, Karlson EW, Cleary PD. Differences between men and women undergoing major orthopedic surgery for degenerative arthritis. Arthritis Rheum 1994;37:687-694.

(127) Keating EM, Meding JB, Faris PM, Ritter MA. Long-term followup of nonmodular total knee replacements. Clin Orthop Relat Res 2002;404:34-39.

(128) Keblish PA, Briard JL. Mobile-bearing unicompartmental knee arthroplasty. J Arthroplasty 2004;19(7 Suppl. 2):87-94.

(129) Keblish PA, Varma AK, Greenwald AS. Patellar resurfacing or retention in total knee arthroplasty. A prospective study of patients with bilateral replacements. J Bone Joint Surg Am 1994;76-B(6):930-937.

(130) Kempshall PJ, Metcalfe A, Forster MC. Review of Kinemax knee arthroplasty performed at the NHS treatment centre, weston-super-mare. J Bone Joint Surg Am 2009;91-B(2):229-233.

(131) Kim KT, Lee S, Park HS, Cho KH, Kim DS. A prospective analysis of Oxford Phase 3 unicompartmental knee arthroplasty. Orthopedics 2007;30(Supplement 1):15-18.

(132) Kim TK, Chang CB, Kang YG, Chung BJ, Cho HJ, Seong SC. Early clinical outcomes of floating platform mobile-bearing TKA: longitudinal comparison with fixed-bearing TKA. Knee Surg Sports Traumatol Arthrosc 2010;18:879-888.

(133) Kim YH, Kim DY, Kim JS. Simultaneous mobile- and fixed-bearing total knee replacement in the same patients. A prospective comparison of mid-term outcomes using a similar design of prosthesis. J Bone Joint Surg Am 2007;89(7):904-910.

(134) Kim YH, Kim JS. Prevalence of osteolysis after simultaneous bilateral fixed- and mobile-bearing total knee arthroplasties in young patients. J Arthroplasty 2009;24:932-940.

(135) Kim YH, Kim JS. Comparison of anterior-posterior-glide and rotating-platform low contact stress mobile-bearing total knee arthroplasties. J Bone Joint Surg Am 2004;86-A:1239-1247.

(136) Kim YH, Kook HK, Kim JS. Comparison of fixed-bearing and mobile-bearing total knee arthroplasties. Clin Orthop Relat Res 2001;392(101-115).

(137) Kim YH, Yoon SH, Kim JS. Early outcome of TKA with a medial pivot fixed-bearing prosthesis is worse than with a PFC mobile-bearing prosthesis. Clin Orthop Relat Res 2009;467:493-503.

(138) Kim YH, Yoon SH, Kim JS. The long-term results of simultaneous fixed-bearing and mobile-bearing total knee replacements performed in the same patient. J Bone Joint Surg Am 2007;89-B(10):1317-1323.

(139) Konig A, Walther M, Kirschner S. Balance sheets of knee and functional scores 5 years after total knee arthroplasty for osteoarthritis: a source for patient information. J Arthroplasty 2000;15(3):289-294.

(140) Kort NP, VanRaay JJ, Cheung J, Jolink C, Deutman R. Analysis of Oxford medial unicompartmental knee replacement using the minimally invasive technique in patients aged 60 and above: an independent prospective series. Knee Surg Sports Traumatol Arthrosc 2007;15(11):1331-1334.

(141) Kort NP, VanRaay JJAM, VanHorn JJ. The Oxford phase III unicompartmental knee replacement in patients less than 60 years of age. Knee Surg Sports Traumatol Arthrosc 2007;15:356-360.

(142) Kramers-deQuervain IA, Engel-Bicik I, Miehlke W, Drobny T, Munzinger U. Fat-pad impingement after total knee arthroplasty with the LCS A/P-Glide system. Knee Surg Sports Traumatol Arthrosc 2005;13:174-178.

(143) Krelbich DN, Vaz M, Bourne RB, Rorabeck CH, Kim P, Hardie R, et al. What is the best way of assessing outcome after total knee replacement? Clin Orthop 1996;331:221-225.

(144) Lampe F, Sufi-Siavach A, Bohlen KE, Hille E, Dries SPM. One year after navigated total knee replacement, no clinically relevant difference found between fixed bearing and mobile bearing knee replacement in a double-blind randomized controlled trial. The Open Orthopaedics Journal 2011;5:201-208.

(145) Langdown AJ, Pandit H, Price AJ, Dodd CAF, Murray DW, Svard UCG, et al. Oxford medial unicompartmental arthroplasty for focal spontaneous osteonecrosis of the knee. Acta Orthop 2005;76(5):688-692.

(146) Larson C, McDowell C, Lachiewicz P. One-peg versus three-peg patella component fixation in total knee arthroplasty. Clin Orthop 2001;392:94-100.

(147) Laurencin CT, Zelicof SB, Scott RD, Ewald FC. Unicompartmental versus total knee arthroplasty in the same patient: A comparative study. Clin Orthop Relat Res 1991;273:151-156.

(148) Lavernia CJ, Guzman JF, Gachupin-Garcia A. Cost effectiveness and quality of life in knee arthroplasty. Clin Orthop 1997;345:134-139.

(149) Leonard L, Williamson DM, Ivory JP, Jennison C. An evaluation of the safety and efficacy of simultaneous bilteral total knee arthroplasty. J Arthroplasty 2003;18(8):972-978.

(150) Li MG, Yao F, Ioppolo J, Nivbrant B, Wood D. Mobile vs. fixed bearing unicondylar knee arthroplasty: A randomized study on short-term clinical outcomes and kee kinematics. Knee 2006;13:365-370.

(151) Liddle AD, Pandit H, O'Brien S, Doran E, Penny ID, Hooper GJ. Cementless fixation in oxford unicompartmental knee replacement; a multicentre study of 1000 knees. Bone Joint J 2013;95-B(2):181-187.

(152) Lim HC, Bae JH, Song SH, Kim SJ. Oxford phase 3 unicompartmental knee replacement in Korean patients. J Bone Jt Surg Br 2012;94(8):1071-1076.

(153) Lingard EA, Katz JN, Wright RJ, Wright EA, Sledge CB. Validity and responsiveness of the knee society clincal rating system in comparison with the SF-36 and WOMAC. J Bone Joint Surg Am 2001;83:1856-1864.

(154) Lingard EA, Wright EA, Sledge CB. Pitfalls of using patient recall to derive preoperative status in outcome studies of total knee arthroplasty. J Bone Joint Surg Am 2001;83:1149-1156.

(155) Lisowski LA, VanDenBekerom MP, Pilot P, VanDijk CN, Lisowski AE. Oxford phase 3 unicompartmental knee arthroplasty: medium-term results of a minimally invasive surgical procedure. Knee Surg Sports Traumatol Arthrosc 2011;19(2):277-284.

(156) Lisowski LA, Verheijen PM, Lisowski AE. Oxford phase 3 unicompartmental knee arthroplasty (UKA): Clinical and radiological results of minimum follow-uip of 2 years. Ortopedia Traumatologia Rehabilitacja 2004;6(6):773-776.

(157) Liu T, Chen S. Simultaneous bilateal total knee arthrooplasty in a single procedure. Int Orthop 1998;22(6):390-393.

(158) Lizaur-Utrilia A, Sanz-Reig J, Trigueros-Rentero MA. Greater satisfaction in older patients with a mobile-bearing compared with fixed-bearing total knee arthroplasty. J Arthroplasty 2012;27(2):207-211.

(159) Lombardi AJ, Mallory T, Fada R. An algorithm for the posterior cruciate ligament in total knee arthroplasty. Clin Orthop 2001;392:75-87.

(160) Lombardi AV, Berend KR, Walter CA, Aziz-Jacobo J, Cheney NA. Is recovery faster for mobile-bearing unicompartmental than total knee arthroplasty? Clin Orthop Relat Res 2009;467:1450-1457.

(161) Lonner JH, Klotz M, Levitz C, Lotke PA. Changes in bone density after cemented total knee arthroplasty. Influence of stem design. J Arthroplasty 2001;16(1):107-111.

(162) Luscombe KL, Lim J, Jones PW, White SH. Minimally invasvie oxford medial unicompartmental knee arthroplasty. A note of caution! Int Orthop 2007;31(3):321-324.

(163) Lyback CO, Belt EA, Hamalainen MMJ, Kauppi MJ, Savolainen HA, Lehto MUK. Survivorship of AGC knee replacement in juvenile chronic arthritis. 13-year follow-up of 77 knees. J Arthroplasty 2000;15(2):166-170.

(164) Lyback CO, Lehto MUK, Hamalainen MMJ, Belt EA. Patellar resurfacing reduces pain after TKA for juvenile rheumatoid arthritis. Clin Orthop Relat Res 2004;423:152-156.

(165) Mackinnon J, Young S, Baily RAJ. The St Georg sledge for unicompartmenal replacement of the knee. A prospective study of 115 cases. J Bone Joint Surg Am 1988;70-B(2):217-223.

(166) March LM, Cross M, Tribe KL, Lapsley HM, Courtenay BG, Cross MJ. Two knees or not two knees? Osteoarthritis Cartilage 2004;12:400-408.

(167) Martin S, McManus J, Scott R. Press-fit condylar total knee arthroplasty. 5- to 9-year follow-up evaluation. J Arthroplasty 1997;12(6):603-614.

(168) Maruyama S, Yoshiya S, Matsui N, Kuroda R, Kurosaka M. Functional comparison of posterior cruciate-retaining versus posterior stabilized total knee arthroplasty. J Arthroplasty 2004;19(3):349-353.

(169) Matharu G, Robb C, Baloch K, Pynsent P. The Oxford medial unicompartmental knee replacement: survival and the affect of age and gender. Knee 2012;19(6):913-917.

(170) Matsueda M, Gustilo R. Subbastus and medial parapatellar approaches in total knee arthroplasty. Clin Orthop 2000;371:161-168.

(171) Mayman D, Bourne RB, Rorabeck CH, Vaz M, Kramer J. Resurfacing versus not resurfacing the patella in total knee arthroplast: 8- to 10-year results. J Arthroplasty 2003;18(5):541-545.

(172) McCaskie AW, Deehan DJ, Green TP, Lock KR, Thompson JR, Harper WM, et al. Randomised, prospective study comparing cemented and cementless total knee replacement.  Results of press-fit condylar total knee replacement of five years. J Bone Joint Surg Am 1998;80-B(6):971-975.

(173) McGuigan FX, Hozack WJ, Moriarty L, Eng K, Rothman RH. Predicting quality-of-life outcomes following total joint arthroplasty. Limitations of the SF-36 health status questionnaire. J Arthroplasty 1995;10:742-747.

(174) Meding J, Ritter M, Faris P. Does the preoperative radiographic degree of osteoarthritis correlate to results in primary total knee arthroplasty? J Arthroplasty 2001;16(1):13-16.

(175) Meding JB, Reddleman K, Keating ME, Klay A, Ritter MA, Faris PM. Total knee replacement in patients with diabetes mellitus. Clin Orthop 2003;416:208-216.

(176) Meding JB, Ritter MA, Faris PM. Total knee arthroplasty with 4.4 mm of tibial polyethylene. Clin Orthop Relat Res 2001;388:112-117.

(177) Mikulak SA, Mahoney OJ, DelaRosak MA, Schmalzried TP. Loosening and osteolysis with the press-fit condylar posterior-cruciate-substituting total knee replacement. J Bone Joint Surg Am 2001;83(3):398-403.

(178) Miner AL, Lingard EA, Wright EA, Sledge CB, Katz JN. Knee range of motion after total knee arthroplasty: how important is this as an outcome measure? J Arthroplasty 2003;18(3):286-294.

(179) Miyasaka K, Ranawat C, Mullaji A. A 10- to 20-year followup of total knee arthroplasty for valgus deformities. Clin Orthop 1997;345:29-37.

(180) Mokris J, Smith S, Anderson S. Primary total knee arthroplasty using the Genesis total knee arthroplasty system: 3- to 6-year follow-up study of 105 knees. J Arthroplasty 1997;12(1):91-98.

(181) Mont M, Yoon T, Krackow K. Eliminating patellofemoral complications in total knee arthroplasty: clinical and radiographic results of 121 consecutive cases using the Duracon system. J Arthroplasty 1999;14(4):446-455.

(182) Morgan SS, Bonshahi A, Pradhan N, Gregory A, Gambhir A, Porter ML. The influence of postoperative coronal alignment on revision surgery in total knee arthroplasty. Int Orthop 2008;32:639-642.

(183) Moskal J, Diduch D. Postoperative radiographs after total knee arthroplasty: a cost-containment strategy. Am J Knee Surg 1998;11(2):89-93.

(184) Mullaji AB, Shetry GM, Kann R. Postoperative limb alignment and its determinants after minimally invasive oxford medial unicompartmental knee arthroplasty. J Arthroplast 2011;26(6):919-925.

(185) Munro JT, Pandit S, Walker CG, Clarworthy M, Pitto RP. Loss of tibial bone density in patients with rotating- or fixed-platform TKA. Clin Orthop Relat Res 2010;468:775-781.

(186) Munzinger UK, Petrich J, Boldt JG. Patella resurfacing in total knee arthroplasty using metal-backed rotating bearing components: a 2- to 10-year follow-up evaluation. Knee Surg Sports Traumatol Arthrosc 2001;9(Suppl 1):S34-S42.

(187) Murray DW, Frost SJD. Pain in the assessment of total knee replacement. J Bone Joint Surg Am 1998;80-B(3):426-431.

(188) Murray DW, Goodfellow JW, O'Connor JJ. The Oxford medial unicompartmental arthroplasty. J Bone Joint Surg Am 1998;80-B(6):983-989.

(189) Nafei A, Nielsen S, Kristensen O, Hvid I. The press-fit kinemax knee arthroplasty. High failure rate of non-cemented implants. J Bone Joint Surg Am 1992;74-B(2):243-246.

(190) Nerhus TK, Heir S, Svege I, Skramm I, Jervidalo T, Madsen JE. Time-dependent improvement in fuctional outcome following oxford medial unicompartmental knee arthroplasty. A prospective longitudinal multicenter study involving 96 patients. Acta Orthop 2012;83(1):46-52.

(191) Newman J, Pydisetty RV, Ackroyd C. Unicompartmental or total knee replacement: the 15-year results of a prospective randomised controlled trial. J Bone Joint Surg Br 2009;91(1):52-57.

(192) Nielsen PT, Hansen EB, Rechnagel K. Cementless total knee arthroplasty in unselected cases of osteoarthritis and rheumatoid arthritis. J Arthroplasty 1992;7(2):137-143.

(193) O'Rourke M, Callaghan J, Goetz D. Osteolysis associated with a cemented modular posterior-cruciate-substituting total knee design: five to eight-year follow-up. J Bone Jt Surg Am 2002;84-A(8):1362-1371.

(194) Otte KS, Larsen H, Jensen TT, Hansen EMB, Rechnagel K. Cementless AGC revision of unicompartmental knee arthroplasty. J Arthroplasty 1997;12(1):55-59.

(195) Pagano MW, Trousdale RT, Stuart MJ, Hanssen AD, Jacofsky DJ. Rotating platform knees did not improve patellar tracking: a prospective, randomized study of 240 primary total knee arthroplasties. Clin Orthop 2004;428:221-227.

(196) Pandit H, Jenkins C, Baker K, Dodd CAF, Murray DW. The Oxford medial unicompartmental knee replacement using a minimally-invasive approach. J Bone Joint Surg Am 2006;88-B(1):54-60.

(197) Pandit H, Jenkins C, Gill HS, Barker K, Dodd CA, Murray DW. Minimally invasive oxford phase 3 unicompartmental knee replacment: results of 1000 cases. J Bone Jt Surg Br 2011;93(2):198-204.

(198) Pandit H, Liddle AD, Kendrick BJ, Jenkins C, Price AJ, Gill HS. Improved fixation in cementless unicompartmental knee replacement: five-year results of a randomized controlled trial. J Bone Joint Surg Am 2013;95(15):1365-1372.

(199) Parsch D, Kruger M, Moser MT, Geiger F. Follow-up of 11-16 years after modular fixed-bearing TKA. Int Orthop 2009;33:431-435.

(200) Pereira D, Jaffe F, Ortiguera C. Posterior cruciate ligament-sparing versus posterior cruciate ligament-sacrificing arthroplasty. Functional results using the same prosthesis. J Arthroplasty 1998;13(2):138-144.

(201) Pitson D, Bhaskaran V, Bond H, Yarnold R, Drewett R. Effectiveness of knee replacement surgery in arthritis. Int J Nurs Stud 1994;31:49-56.

(202) Price AJ, Dodd CAF, Svard UGC, Murray DW. Oxford medial unicompartmental knee arthroplasty in patients younger and older than 60 years of age. J Bone Joint Surg Am 2005;87-B(11):1488-1492.

(203) Price AJ, Rees JL, Beard D, Juszczak E, Carter S, White S. A mobile-bearing total knee prosthesis compared with a fixed-bearing prosthesis; a multicentre single blind randomised controlled trial. J Bone Joint Surg Br 2003;85-B(1):62-67.

(204) Pynsent PB, Adams DJ, Disney SP. The Oxford hip and knee outcome questionnaires for arthroplasty. J Bone Joint Surg Br 2005;87(2):241-248.

(205) Rajasekbar C, Das S, Smith A. Unicompartment knee arthroplasty 2- to 12- year results in a community hospital. J Bone Joint Surg Am 2004;86-B(7):983-985.

(206) Rand JA. Cement or cementless fixation in total knee arthroplasty? Clin Orthop Relat Res 1991;273:52-62.

(207) Rand JA, Trousdale RT, Ilstrup DM, Harmsen WS. Factors affecting the durability of primary total knee prosthesis. J Bone Joint Surg Am 2003;85-A(2):259-265.

(208) Rasquinha VJ, Ranawat CS, Cervieri CL, Rodriguez JA. The press-fit condylar modular total knee system with a posterior cruciate-substituting design. J Bone Joint Surg Am 2006;88-A(5):1006-1010.

(209) Reay E, Wu J, Holland J, Deehan D. Premature failure of kinemax plus total knee replacements. J Bone Joint Surg Am 2009;91-B(5):604-611.

(210) Rees JL, Price AJ, Beard DJ, Dodd CA, Murray DW. Minimally invasive oxford unicompartmental knee arthroplasty: functional results at 1 year and the effect of surgical inexperience. Knee 2004;11(5):363-367.

(211) Regner L, Carlsson L, Karrholm J. Clinical and radiologic survivorship of cementless tibial components fixed with finned polyethylene pegs. J Arthroplasty 1997;12(7):751-758.

(212) Rinta-Kiikka I, Savilahti S, Pajamaki J. A five to seven years follow-up of 102 cementless synatomic knee arthroplasties. Ann Chir Gynaecol 1996;85(1):77-85.

(213) Rinta-Kiikka I, Savilahti S, Pajamaki J, Lindholm TS. Intermediate-term clinical and radiographic results of synatomic and AGC knee prostheses. Orthopedics 1999;22(3):295-299.

(214) Ritter M, Worland R, Saliski J. Flat-on-flat, nonconstrained, compression molded polyethylene total knee replacement. Clin Orthop 1995;321:79-85.

(215) Ritter MA, Berend ME, Meding JB, Keating EM, Faris PM, Crites BM. Long-term followup of anatomic graduated components posterior cruciate-retaining total knee replacement. Clin Orthop Relat Res 2001;388:51-57.

(216) Ritter MA, Harty LD, Davis KE, Meding JB, Berend M. Simultaneous bilateral, staged bilateral, and unilateral total knee arthroplasty. A survival analysis. J Bone Joint Surg Am 2003;85-A(8):1532-1537.

(217) Ritter MA, Wing JT, Berend ME, Davis KE, Meding JB. The clinical effect of gender on outcome of total knee arthroplasty. J Arthroplasty 2008;23(3):331-336.

(218) Rodricks DJ, Patil S, Pulido P, Colwell CWJ. Press-fit condylar design total knee arthroplasty. Fourteen to Seventeen-year follow-up. J Bone Joint Surg Am 2007;89-A(1):89-95.

(219) Rodriguez J, Saddler S, Edelman S. Long-term results of total knee arthroplasty in class 3 and 4 rheumatoid arthritis. J Arthroplasty 1996;11(2):141-145.

(220) Rodriguez JA, Baez N, Rasquinha V, Ranawat CS. Metal-backed and all-polyethylene tibial components in total knee replacement. Clin Orthop Relat Res 2001;392:174-183.

(221) Rosenberg N, Henderson I. Medium term outcome of the LCS cementless posterior cruciate retaining total knee replacements. Follow up and survivorship study of 35 operated knees. The Knee 2001;8:123-128.

(222) Sanchez-Sotelo J, Ordonez JM, Prats SB. Results and complications of the low contact stress knee prosthesis. J Arthroplasty 1999;14(7):815-821.

(223) Sansone V, daGama MM. Mobile-bearing total knee prosthesis: a 5- to 9-year follow-up of the first 110 consecutive arthroplasties. J Arthroplasty 2004;19(6):678-685.

(224) Santini AJA, Raut V. Ten-year survival analysis of the PFC total knee arthroplasty - a surgeon's first 99 replacements. Int Orthop 2008;32:459-465.

(225) Schai PA, Thornhill TS, Scott RD. Total knee arthroplasty with the PFC system. Results at a minimum of ten years and survivorship analysis. J Bone Joint Surg Am 1998;80-B(5):850-858.

(226) Schelfaut S, Beckers L, Verdonk P, Bellemans J, Victor J. The risk of of bearing dislocation in lateral unicompartmental knee arthroplasty using a mobile biconcave design. Knee Surg Sports Traumatol Arthrosc 2012.

(227) Schroder H, Berthelsen A, Hassan G. Cementless porous-coated total knee arthroplasty: 10-year results in a consecutive series. J Arthroplasty 2001;16(5):559-567.

(228) Schroder HM, Aaen K, Hansen EB, Nielsen PT, Rechnagel K. Cementless total knee arthroplasty in rheumatoid arthritis. A report on 51 AGC knees followed for 54 months. J Arthroplasty 1996;11(1):18-23.

(229) Sextro G, Berry D, Rand J. Total knee arthroplasty using cruciate-retaining kinematic condylar prosthesis. Clin Orthop 2001;388:33-40.

(230) Sharma L, Sinacore J, Daugherty C, Kuesis DT, Stulberg SD, Lewis M, et al. Prognostic factors for functional outcome of total knee replacement: a prospective study. J Gerontol A Biol Sci Med Sci 1996;61:M152-7.

(231) Sharma S, Nicol F, Hullin MG, McCreath SW. Long-term results of the uncemented low contact stress total knee replacement in patients with rheumatoid arthritis. J Bone Joint Surg Am 2005;87-B(8):1077-1080.

(232) Shih HN, Shih LY, Wong CY, Hsu RW. Long-term changes of the nonresurfaced patella after total knee arthroplasty. J Bone Joint Surg Am 2004;86-A:935-939.

(233) Song MH, Kim BH, Ahn SJ, Yoo SH, Lee MS. Early complications after minimally invasive mobile-bearing medial unicompartmental knee arthroplasty. J Arthoplast 2009;24(8):1281-1284.

(234) Sorrells RB. Primary knee arthroplasty: Long-term outcomes. The rotating platform mobile bearing TKA. Orthopedics 1996;19(9):793-796.

(235) Sorrells RB, Stiehl JB, Voorhorst PE. Midterm results of mobile-bearing total knee arthroplasty in patients younger than 65 years. Clin Orthop Relat Res 2001;390:182-189.

(236) Sorrells RB, Voorhorst PE, Murphy JA, Bauschka MP, Greenwald AS. Uncemented rotating-platform total knee replacement; a five to twelve-year follow-up study. J Bone Joint Surg Am 2004;86-A:2156-2162.

(237) Starks I, Roberts S, White SH. The Avon patellofemoral joint replacement: independent assessment of early functional outcomes. J Bone Joint Surg Br 2009;91(12):1579-1582.

(238) Stickless B, Phillips L, Brox W. Defining the relationship between obesity and total joint arthroplasty. Obes Res 2001;9(3):219-223.

(239) Stockley I, Douglas DL, Elson RA. Bicondylar St. Georg sledge knee arthroplasty. Clin Orthop Relat Res 1990;255:228-234.

(240) Stukenborg-Colsman C, Wirth CJ, Lazovic D, Wefer A. High tibial osteotomy versus unicompartmental joint replacement in unicompartmental knee joint osteoarthritis: 7-10-year follow-up prospective randomised study. Knee 2001;8(3):187-194.

(241) Sun PF, Jia YH. Mobile bearing UKA compared to fixed bearing TKA: a randomized prospective study. Knee 2012;19(2):103-106.

(242) Svard UCG, Price AJ. Oxford medial unicompartmental knee arthroplasty. A survival analysis of an independent series. J Bone Joint Surg Am 2001;83-B(2):191-194.

(243) Tarkin IS, Bridgeman JT, Jardon OM, Garvin KL. Successful biologic fixation with mobile-bearing total knee arthroplasty. J Arthroplasty 2005;20(4):481-486.

(244) Therbo M, Petersen MM, Varmarken JE, Olsen CA, Lund B. Influence of pre-operative bone mineral content of the proximal tibia on revision rate after uncemented knee arthroplasty. J Bone Joint Surg Am 2003;85-B(7):975-979.

(245) Tibesku CO, Daniilidis K, Vieth V, Skwara A, Heindel W, Fuchs-Winkelmann S. Sagittal plane kinematics of fixed- and mobile-bearing total knee replacements. Knee Surg Sports Traumatol Arthrosc 2011;19:1488-1495.

(246) Title C, Rodriguez J, Ranawat C. Posterior cruciate-sacrificing versus posterior cruciate-substituting total knee arthroplasty; a study of clinical and fuctional outcomes in matched patients. J Arthroplasty 2001;16(4):409-414.

(247) Verdonk R, Cottenie D, Almqvist KF, Vorlat P. The Oxford unicompartmental knee prosthesis: a 2-14 year follow-up. Knee Surg Sports Traumatol Arthrosc 2005;13:163-166.

(248) Ververeli P, Sutton D, Hearn S. Continuous passive motion after total knee arthroplasty. Analysis of cost and benefits. Clin Orthop 1995;321:208-215.

(249) Vessely MB, Whaley AL, Harmsen WS, Schleck CD, Berry DJ. Long-term survivorship and failure modes of 1000 cemented condylar total knee arthroplasties. Clin Orthop Relat Res 2006;452:28-34.

(250) Vorlat P, Putzeys G, Cottenie D, VanIsacker T, Pouliart N, Handelberg F, et al. The Oxford unicompartmental knee prosthesis: an independent 10-year survival analysis. Knee Surg Sports Traumatol Arthrosc 2006;14:40-45.

(251) Vorlat P, Verdonk R, Schauvlieghe H. The Oxford unicompartmental knee prosthesis: a 5-year follow-up. Knee Surg Sports Traumatol Arthrosc 2000;8:154-158.

(252) Watanabe T, Tomica T, Fujii M, Hashimoto J, Sugamoto K, Yoshikawa H. Comparison between mobile-bearing and fixed-bearing knees in bilateral total knee replacements. Int Orthop 2005;29(3):179-181.

(253) Waters TS, Bentley G. Patellar resurfacing in total knee arthroplasty. A prospective, randomized study. J Bone Joint Surg Am 2003;85-A(2):212-217.

(254) Weale AE, Newman JH. Unicompartmental arthroplasty and high tibial osteotomy for osteoarthritis of the knee: A comparative study with a 12- to 17-year follow-up period. Clin Orthop Relat Res 1994;302:134-137.

(255) Weaver JK, Derkash RS, Greenwald AS, Oxon DP. Difficulities with bearing dislocation and breakage using a movable bearing total knee replacement system. Clin Orthop Relat Res 1993;290:244-252.

(256) Weber AB, Worland RL, Keenan J, VanBowen J. A study of polyethylene and modularity issues in <1,000 posterior cruciate - retaining knees at 5 to 11 years. J Arthroplasty 2002;17(8):967-991.

(257) Westwood MJ, White SP, Bannister GC. The St.leger total knee replacement.  A false economy? The Knee 2003;10:179-180.

(258) Woolson ST, Northrop GD. Mobile - vs. fixed-bearing total knee arthroplasty. J Arthroplasty 2004;19(2):135-140.

(259) Worland R, Arredondo J, Angles F. Home continuous passive motion machine versus professional physical therapy following total knee replacement. J Arthroplasty 1998;13(7):784-787.

(260) Worland RL, Johnson G, Alemparte J, Jessup DE, Keenan J, Norambuena N. Ten to fourteen year survival and functional analysis of the AGC total knee replacement system. The Knee 2002;9:133-137.

(261) Wright RJ, Lima J, Scott RD, Thornhill TS. Two- to four-year results of posterior cruciate-sparing condylar total knee arthroplasty with an uncemented femoral component. Clin Orthop Relat Res 1990;260:80-86.

(262) Wright RJ, Sledge CB, Poss R, Ewald FC, Walsh ME, Lingard EA, et al. Patient-reported outcome and survivorship after kinemax total knee arthroplasty. J Bone Joint Surg Am 2004;86-A(11):2464-2470.

(263) Yang K, Yeo S, Lee B. Total knee arthroplasty in diabetic patients: a study of 109 consecutive cases. J Arthroplasty 2001;16(1):102-106.

(264) Yang KY, Wang MC, Yeo SJ, Lo NN. Minimally invasive unicondylar versus total condylar knee arthroplasty - early results of a matched-pair comparison. Singapore Med J 2003;44(11):559-562.

(265) Zaki SH, Rafiq I, Kapoor A, Raut V, Gambhir AK, Porter ML. Medium term results with the press fit condylar (PFC) sigma knee prosthesis. The Wrightington experience. Acta Orthop Belg 2007;73:55-59.
